# Supplementary material for: A statistical framework for differential pseudotime analysis with multiple single-cell RNA-seq samples
Source: Nat Commun. 2023 Nov 10;14:7286. doi: 10.1038/s41467-023-42841-y (PMC10638410; doi:10.1038/s41467-023-42841-y)
Supplement: Supplementary file 1 — Supplementary Information [file 41467_2023_42841_MOESM1_ESM.pdf]

Supplementary Figures

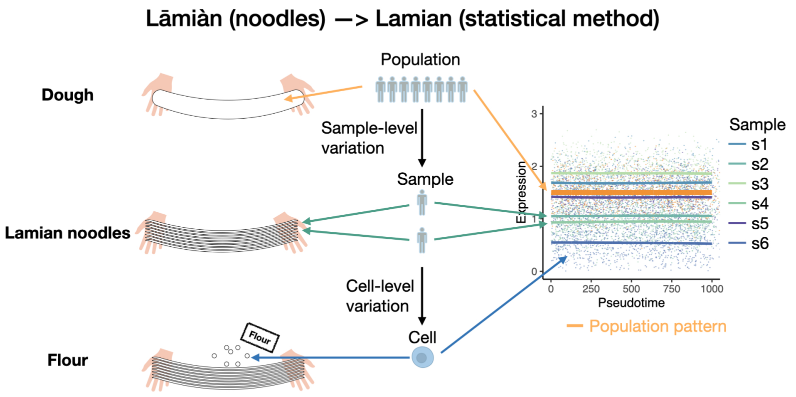

Figure S1. The statistical model is named as `Lamian`, a traditional Chinese hand-pulled noodle, based on the similarity between the model and the process of making Lamian. To make the Lamian noodles, one first makes a firm dough using wheat flour, and then pulls many thinner noodles from the dough. Finally, one coats the noodles with extra flour to prevent the noodles from sticking to each other. In the method `Lamian`, the population-level pattern (the thick orange curve) of gene expression dynamics in all samples resembles the dough. The sample-level gene expression dynamics are modeled as the population-level pattern plus some sample-level random variation, resulting in a curve for each sample (the thin curves). The sample-level curves resemble the thinner noodles coming from the dough. Finally, the observed cell-level gene expression values are modeled as the sample-level curve plus some cell-level random variation. Here the cells resemble the flour.

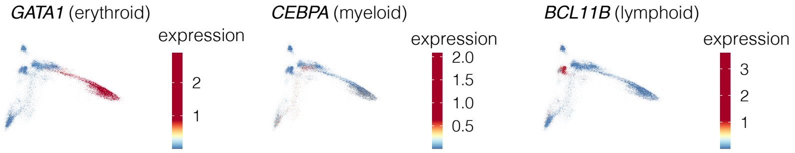

Figure S2. Marker gene expression levels in the three main lineages of hematopoietic stem cell differentiation in HCA-BM data. Source data are provided as a Source Data file.

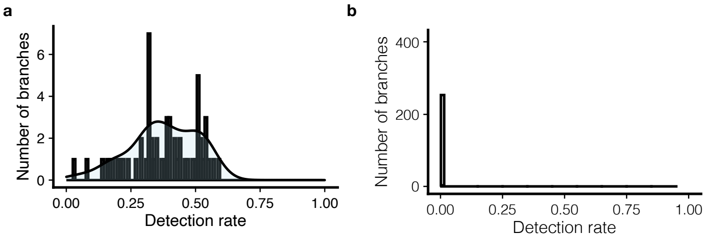

c

Number of clusters	Time (minutes)	Memory(GB)
6 (Fig. 2)	9.183	4.096
100	134.800	4.965
1000	404.650	6.297

Figure S3. Inferring tree structure (Lamian Module 1) in HCA-BM data by setting a large number of cell clusters in TSCAN. (a) Distribution of detection rates of all tree branches when the number of clusters is 100. **(b)** Same as (a) except that the number of clusters is 1000. **(c)** Computational time and memory usage for Lamian Module 1 with different numbers of clusters. Source data are provided as a Source Data file.

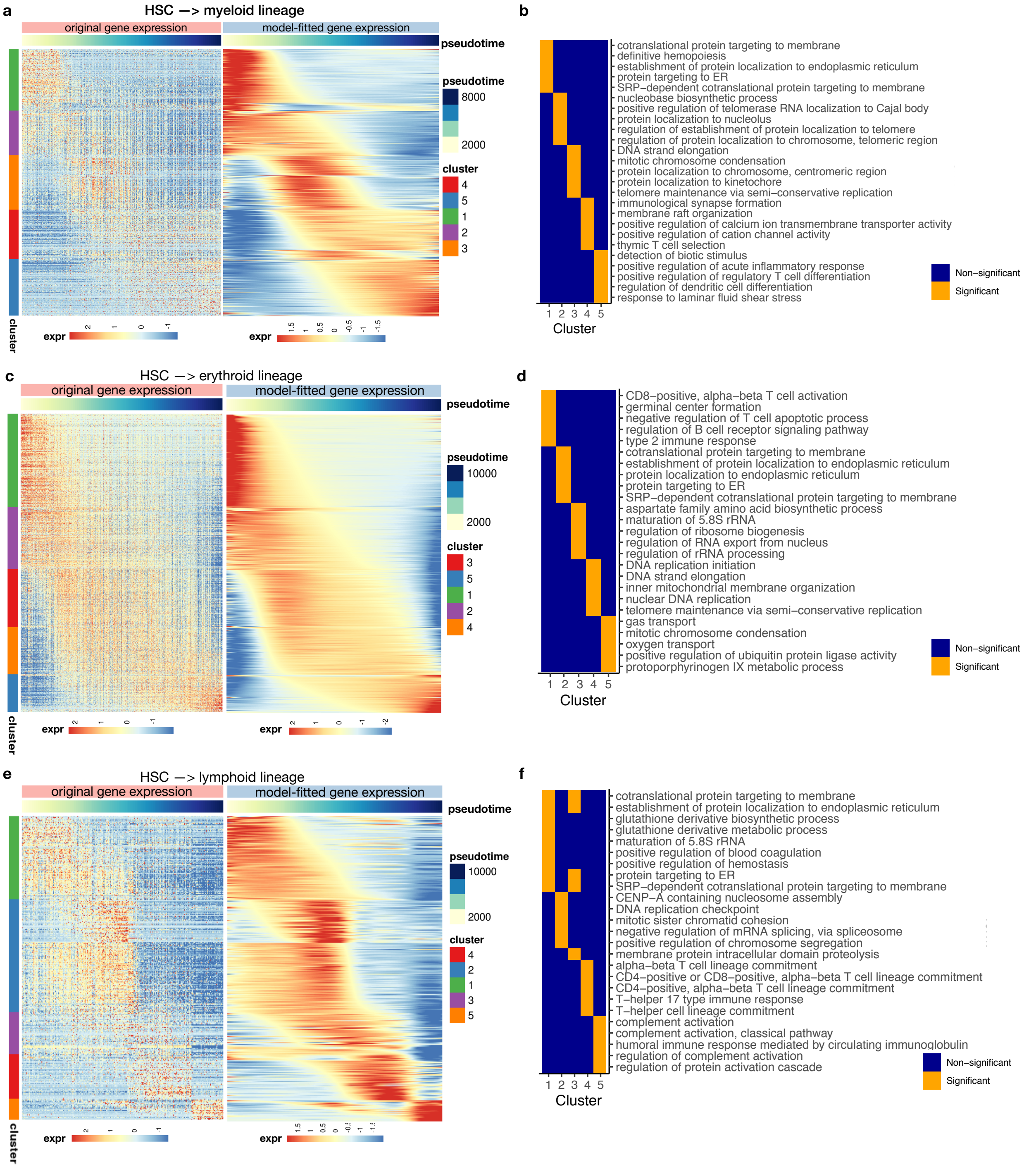

Figure S4. Differentially expressed genes along pseudotime found using Lamian's TDE test (Module 3) along the (a,b) myeloid lineage, (c,d) erythroid lineage, and (e,f) lymphoid lineage in HCA-BM data. (a, c, e) Heatmaps of original gene expression values (left) and model-fitted values (right) along pseudotime. Rows are TDE genes identified by Lamian using a FDR < 0.05 threshold and clustered using *k*-means clustering. Columns are cells ordered by pseudotime. **(b, d, f)** Heatmaps of enriched gene ontology (GO) terms identified using `topGO`(v.2.36.0)[1] package with $FDR < 0.05$ and $FC > 2$ cutoff for the gene clusters shown in (a, c, e), respectively. Source data are provided as a Source Data file.

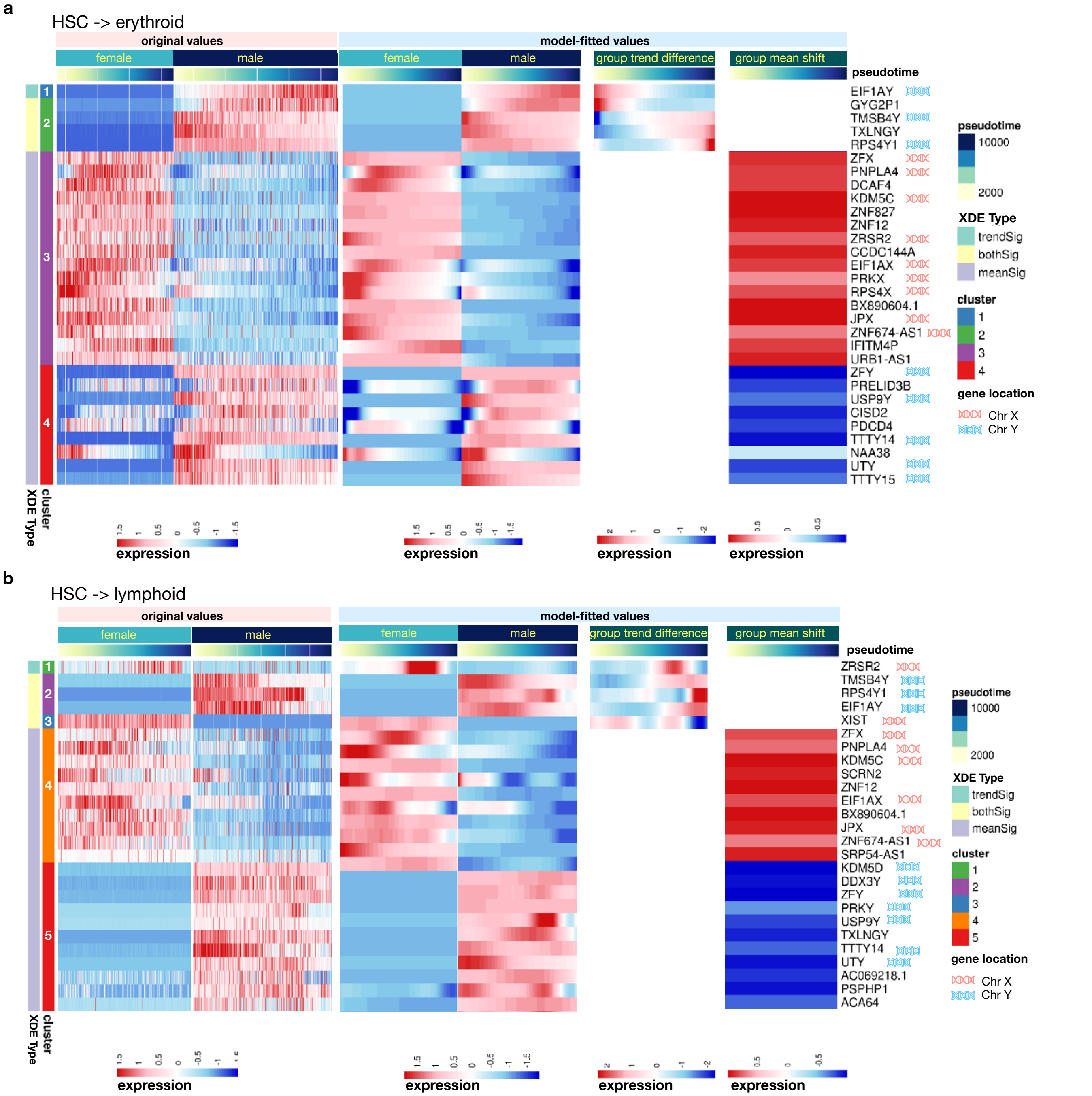

Figure S5. Heatmaps of XDE genes along the (a) erythroid and (b) lymphoid lineages associated with sex (male, female) as a covariate found by Lamian's XDE test (Module 3) in the HCA-BM data. Rows are genes identified using FDR < 0.05. Only XDE genes with significant mean shift, trend difference, or both are shown. In the erythroid lineage, 53% (or n=17) of the XDE genes identified by Lamian are on sex chromosomes (one-sided permutation test p-values for the enrichment of sex chromosome genes: 0.017 for chrX and 0.000 for chrY). In the lymphoid lineage, 62% (or n=18) of the XDE genes identified by Lamian are on sex chromosomes (one-sided permutation test p-values for the enrichment of sex chromosome genes: 0.001 for chrX and 0.000 for chrY). Source data are provided as a Source Data file.

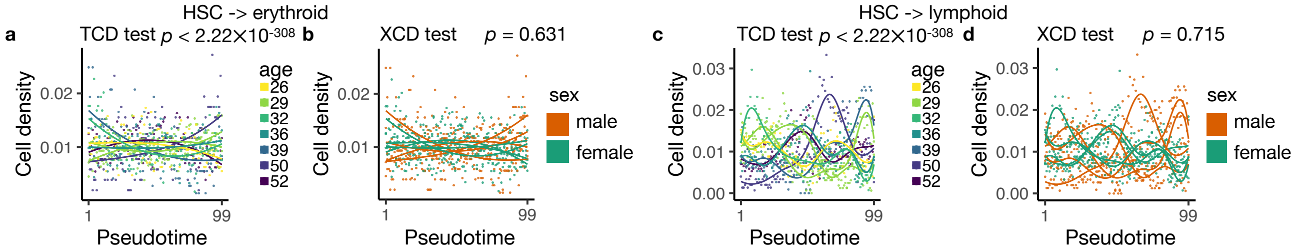

Figure S6. TCD and XCD test (Module 4) results for the HCA-BM data on (a,b) erythroid and (c,d) lymphoid lineages. The pseudotime was split into 100 equally-spaced bins. Each dot represents the cell density in each bin of one sample (i.e., the number of cells in that bin divided by the total number of cells in that sample). Each curve represents the model-fitted temporal pattern of a sample. In TCD test (n = 8, LLR = 113.12 (a) and 196.31 (c)), the curves are colored by individual samples. In XCD test (n = 8, LLR = 2.46 (b) and 6.52 (d)), they are colored by the sample group. One-sided *p*-values are shown. Source data are provided as a Source Data file.

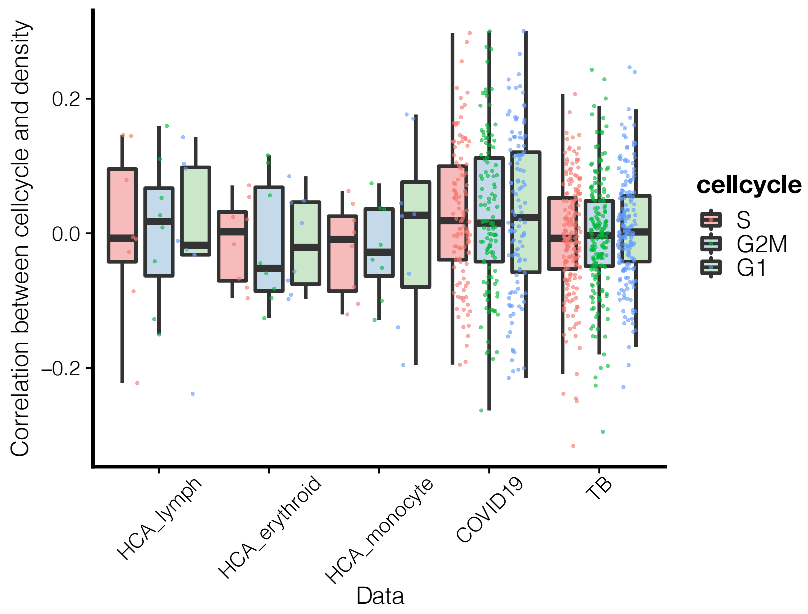

Figure S7. Relationship between cell cycle and cell density along pseudotime. The cell cycle gene lists of S-phase (43 genes) and G2-M-phase (54 genes) were retrieved from Seurat v.3.2.1. The gene list of G1-phase was retrieved from GSEA G1_PHASE (15 genes). For each cell, S-, G2-M- and G1-phase scores were calculated using the average of the normalized log2-transformed gene expression across genes in the corresponding gene lists. In this way, with the cellular pseudotime, the pseudotemporal pattern of the cell cycle was obtained. For each sample, Pearson correlation coefficients between cell cycle scores and cell density along pseudotime were calculated. The boxplots show the distributions (centre: median; bounds of box: 1st and 3rd quartiles; bounds of whiskers: data points within 1.5 IQR from the box; minima; maxima) of correlation coefficients for different datasets and tree branches (each data point represents a sample). The sample sizes are $n = 8$ for each box in HCA, $n = 114$ for each box in COVID19, and $n = 184$ for each box in TB data. Source data are provided as a Source Data file.

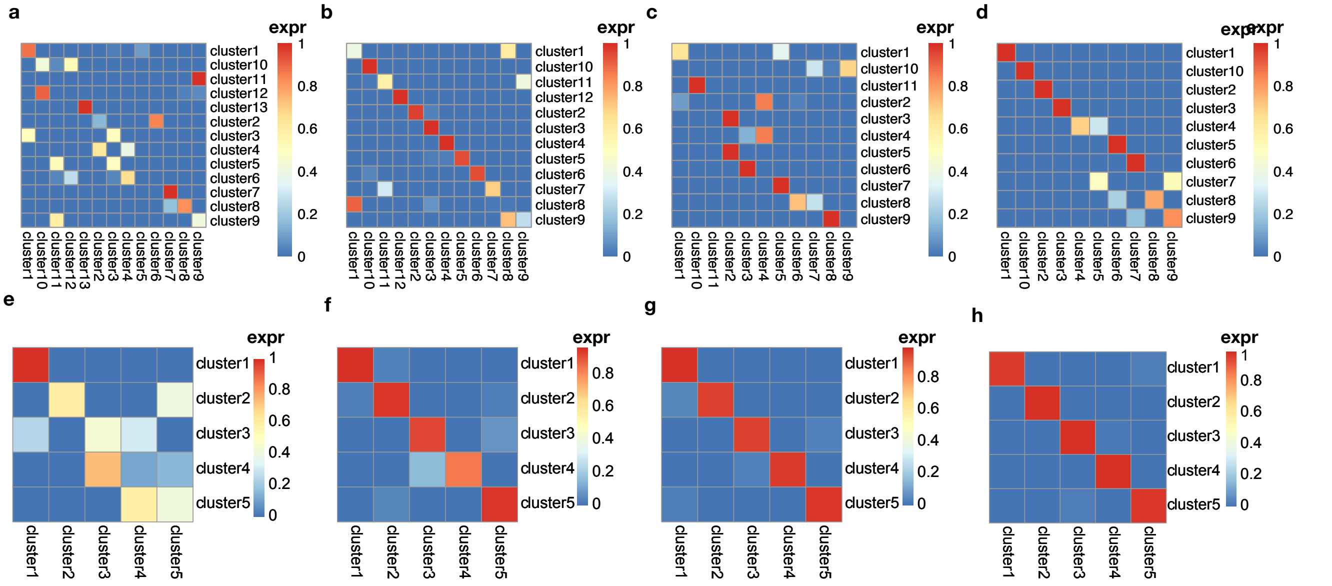

Figure S8. Comparison of *k*-means and Gaussian mixture model clustering results of differential genes. (a)-(d) Clustering XDE genes in the *in silico* spike-in datasets used in Fig. 4b-d. Heatmaps show the confusion matrix of k-means and Gaussian mixture model (GMM) clustering results for data with increasing signal strength ((a) to (d) represents signal strength from 1 to 4, respectively). **(e)-(h)** Clustering TDE genes in the *in silico* spike-in datasets used in Fig. S14. Similar to (a)-(d) but on TDE genes. Overall, the clustering results from the two clustering methods become more similar as the signal strength increases. expr means expression. Source data are provided as a Source Data file.

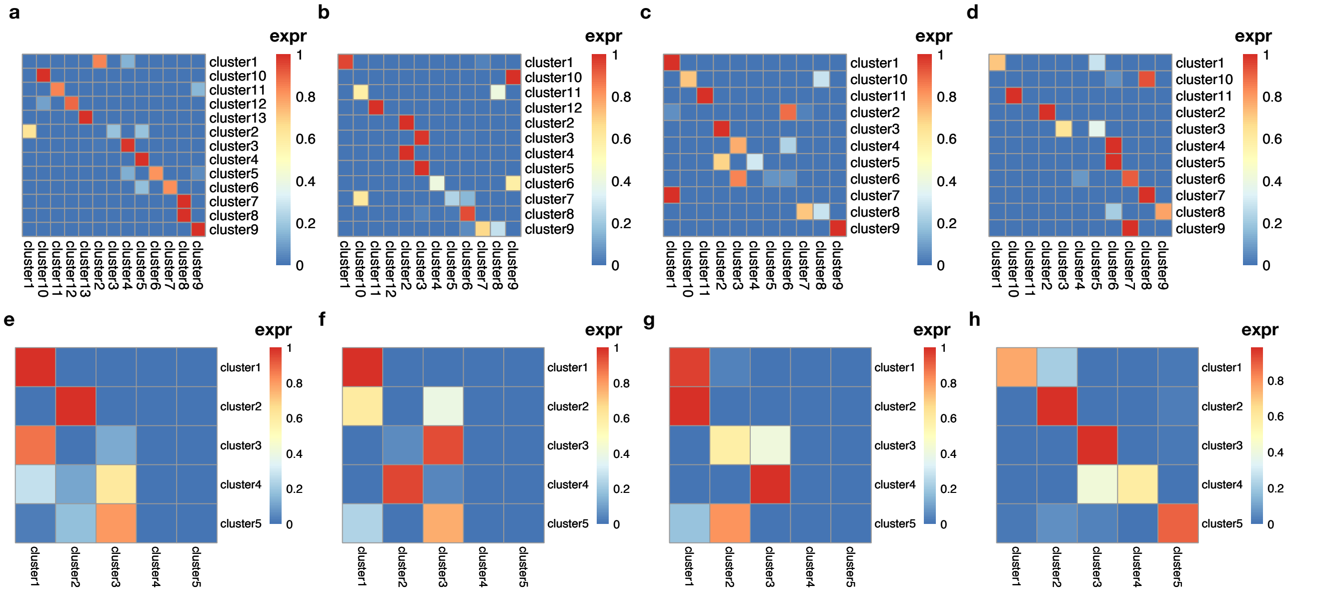

Figure S9. Comparison of *k*-means and Louvain clustering results of differential genes. (a)-(d) Clustering XDE genes in the *in silico* spike-in datasets used in Fig. 4b-d. Heatmaps show the confusion matrix of k-means and Louvain clustering results for data with increasing signal strength ((a) to (d) represents signal strength from 1 to 4, respectively). **(e)-(h)** Clustering TDE genes in the *in silico* spike-in datasets used in Fig. S14. Similar to (a)-(d) but on TDE genes. Overall, the clustering results from the two clustering methods become more similar as the signal strength increases. expr means expression. Source data are provided as a Source Data file.

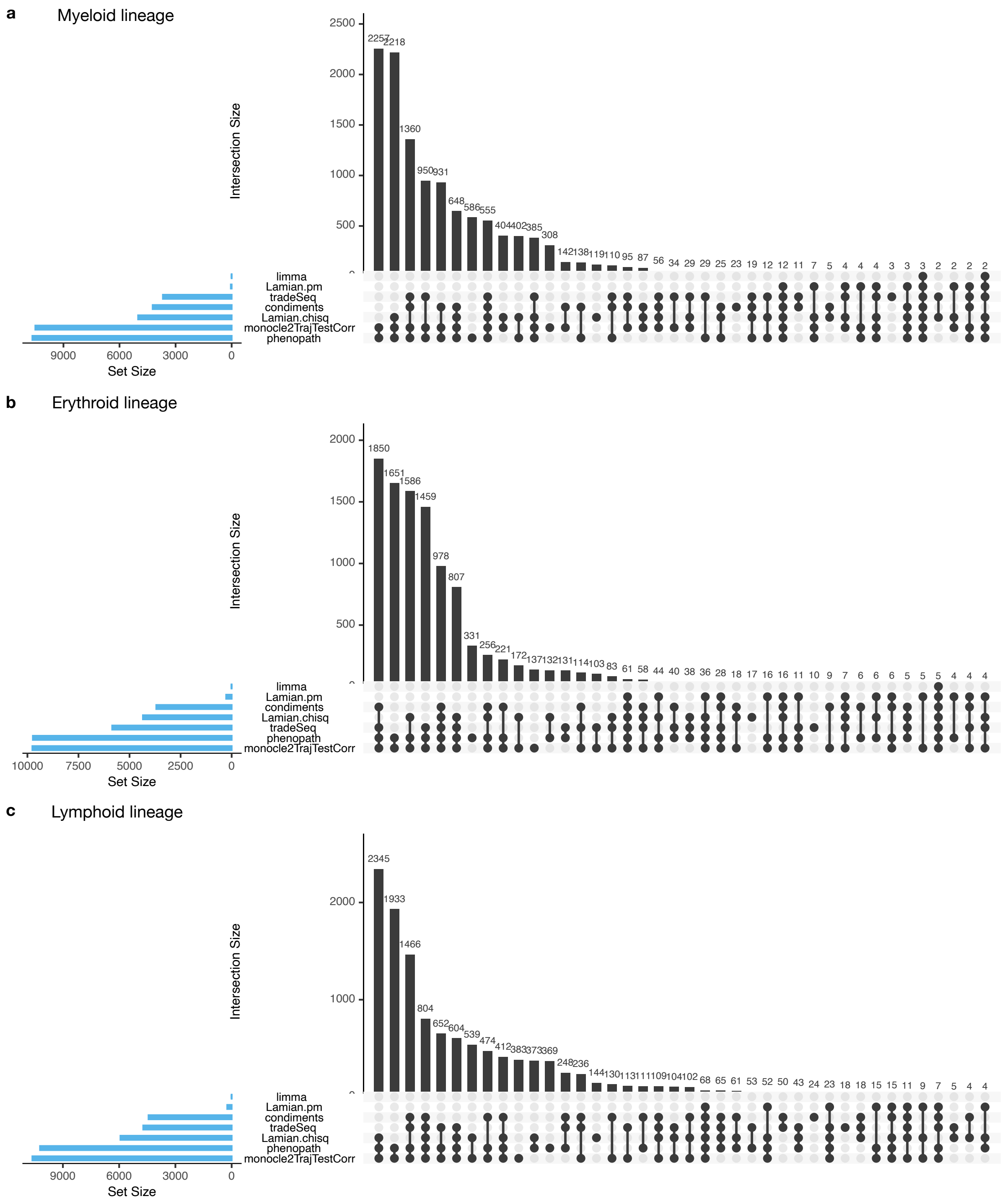

Figure S10. UpSet plots showing the number of XDE genes reported by different methods and their intersections in the HCA-BM data. (a)-(c) represent three differentiation lineages. Source data are provided as a Source Data file.

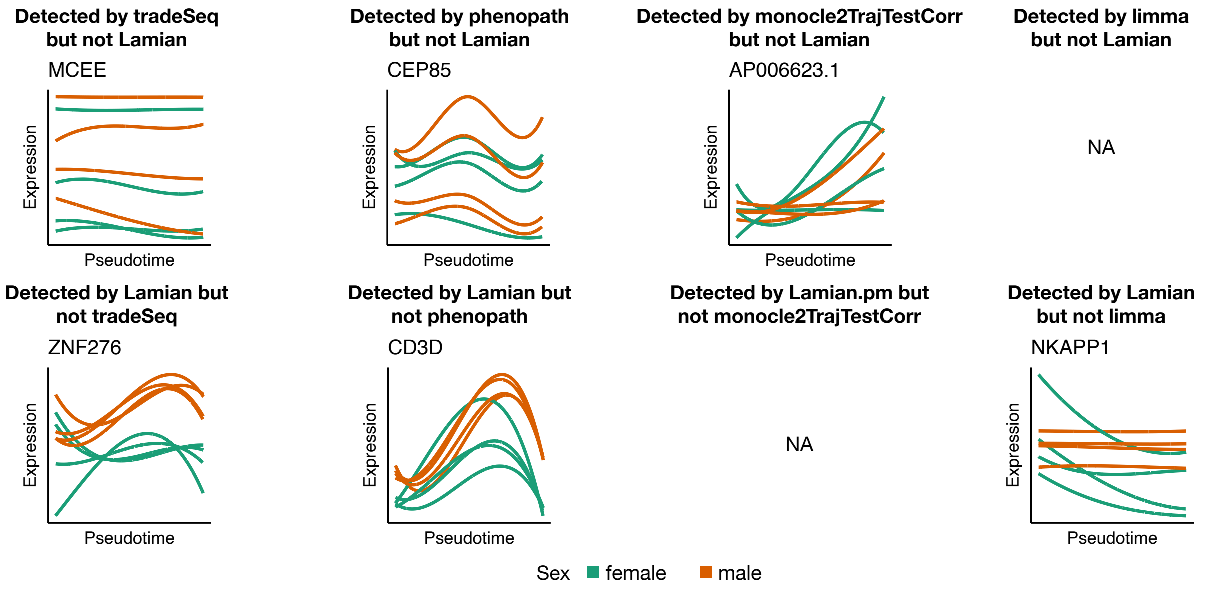

Figure S11. Example XDE genes in HCA-BM data's myeloid lineage. Each column represents a comparison between Lamian and another method. Within the column, the top row shows an example gene detected by the other method but not Lamian, and the bottom row shows an example gene detected by Lamian but not the other method. N/A means no genes are available in the corresponding category. Each plot shows fitted curves for individual samples color-coded by sample group. Source data are provided as a Source Data file.

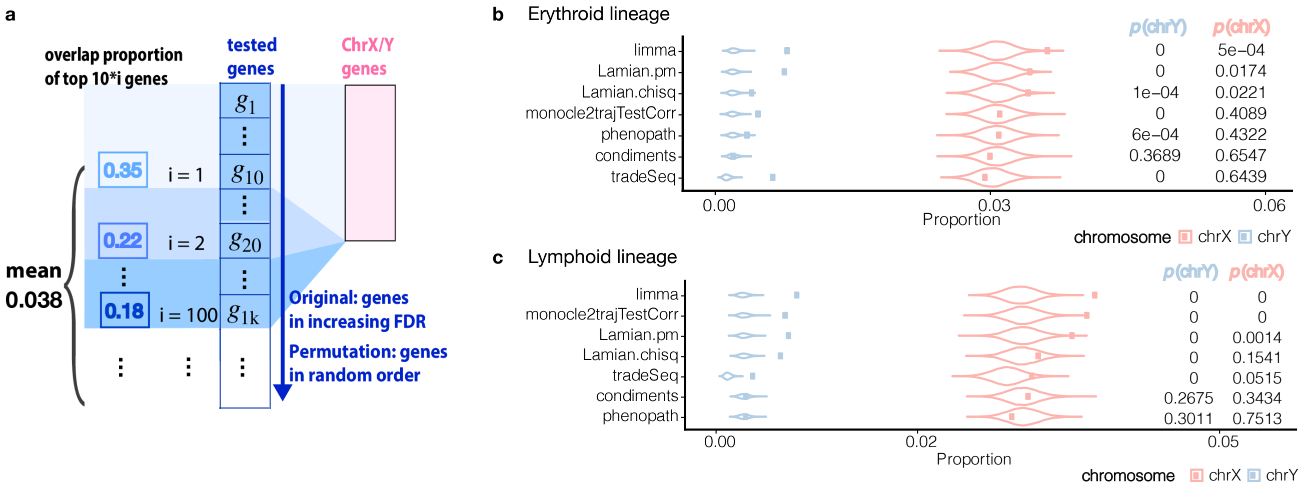

Figure S12. Comparison of sex-associated XDE genes reported by different methods in HCA-BM data. **(a)** Schematic view of the calculation of overlap proportion with sex chromosomes as an evaluation metric in Fig. 4i. **(b-c)** Comparison of XDE genes detected by Lamian and other methods in the erythroid lineage using $n = 8$ samples (b) and lymphoid lineage ($n = 8$) (c). Overlap (dot) between XDE genes reported by different methods and sex chromosome genes as a gold standard, along with permutation test null distribution (violin plot) of the overlap and p-values (one-sided). Source data are provided as a Source Data file.

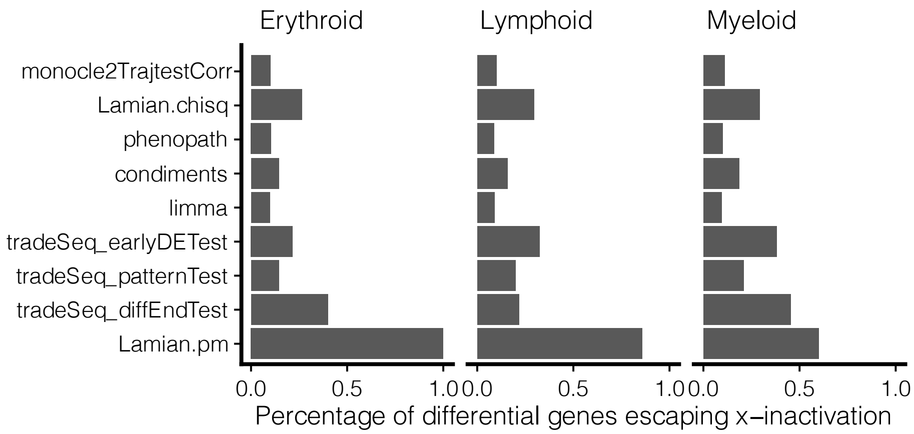

Figure S13. Comparison of Lamian and other methods based on the percentage of sex-associated XDE genes escaping X-chromosome inactivation (XCI) in HCA-BM data. XCI status is obtained from Balaton et al[2]. Source data are provided as a Source Data file.

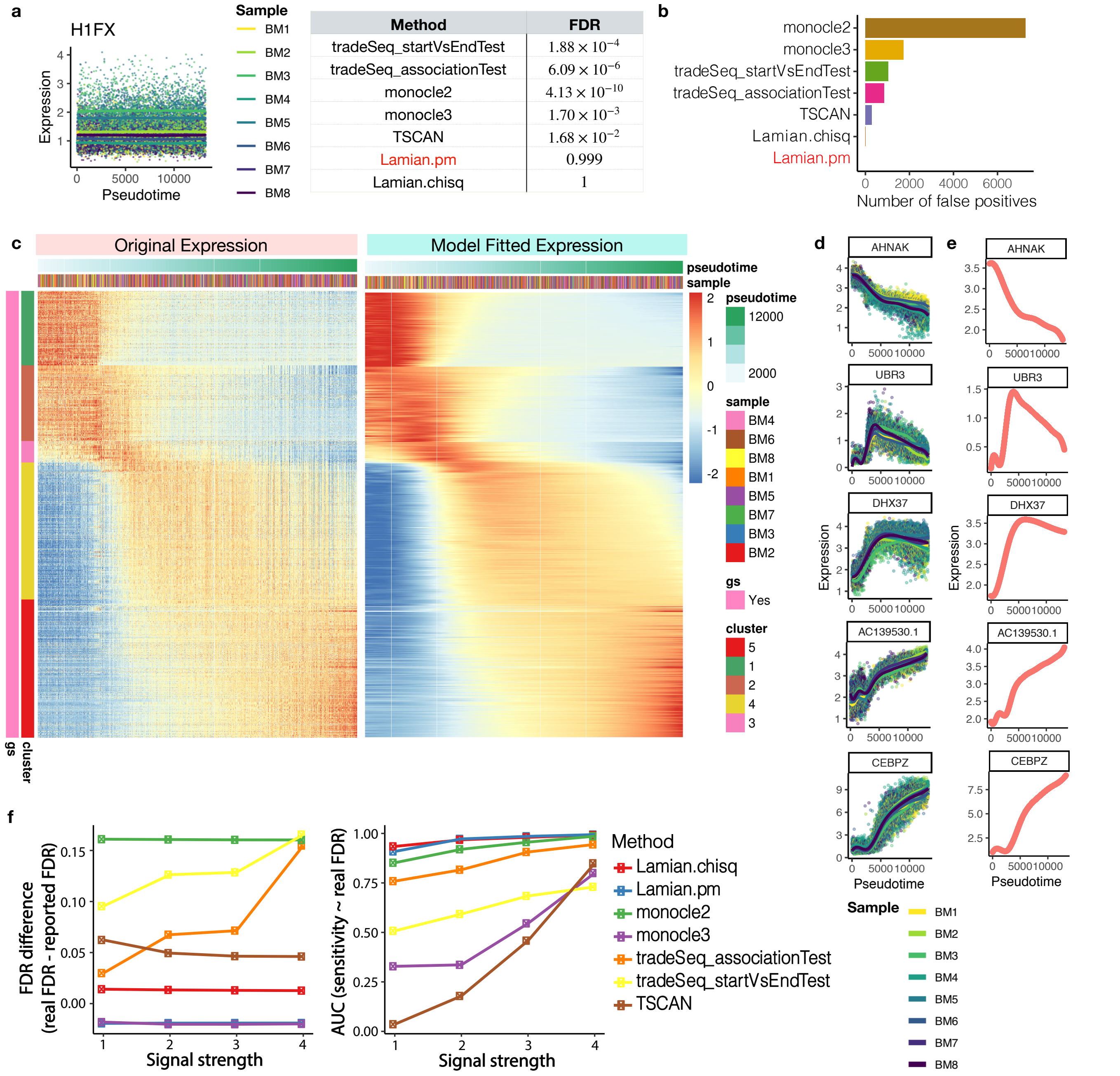

Method	FDR
tradeSeq_startVsEndTest	1.88×10^{-4}
tradeSeq_associationTest	6.09×10^{-6}
monocle2	4.13×10^{-10}
monocle3	1.70×10^{-3}
TSCAN	1.68×10^{-2}
Lamian.pm	0.999
Lamian.chisq	1

Figure S14. Simulation study to assess performance of TDE test. (a-b) Null simulation. **(a)** An example null gene without differential expression along pseudotime (n = 8). Each dot represents a cell colored by the sample it belongs to. The gene's expression (y-axis) in each cell is shown along pseudotime (x-axis). Curves are the model-fitted values for each sample estimated by Lamian. The table shows the FDR reported by Lamian and existing methods about this example gene. **(b)** Number of false positives reported by each method in the null simulation. **(c-f)** Spike-in simulation. **(c)** Heatmap showing expression profiles of TDE genes (rows) along pseudotime-ordered cells (columns) in original values (left) and model-fitted values (right). gs = gold standard TDE genes. Lamian grouped TDE genes into 5 clusters. **(d)** Five example genes for cluster 1 (top) to 5 (bottom) shown in (c). Each dot is a cell showing the SAVER-imputed expression. **(e)** The population-level gene expression estimates along pseudotime. **(f)** Performance evaluation. The left plot shows the difference between the true and reported FDR. A negative value indicates that the reported FDR properly controls the real FDR. A positive value indicates that the reported FDR underestimates the real FDR. The right plot shows the area under the sensitivity-realFDR curve (AUC). The larger the AUC, the more powerful a method is. `PseudotimeDE` did not provide output with 400GB and 20 CPU cores in parallel within 7 days and therefore is not shown here. Source data are provided as a Source Data file.

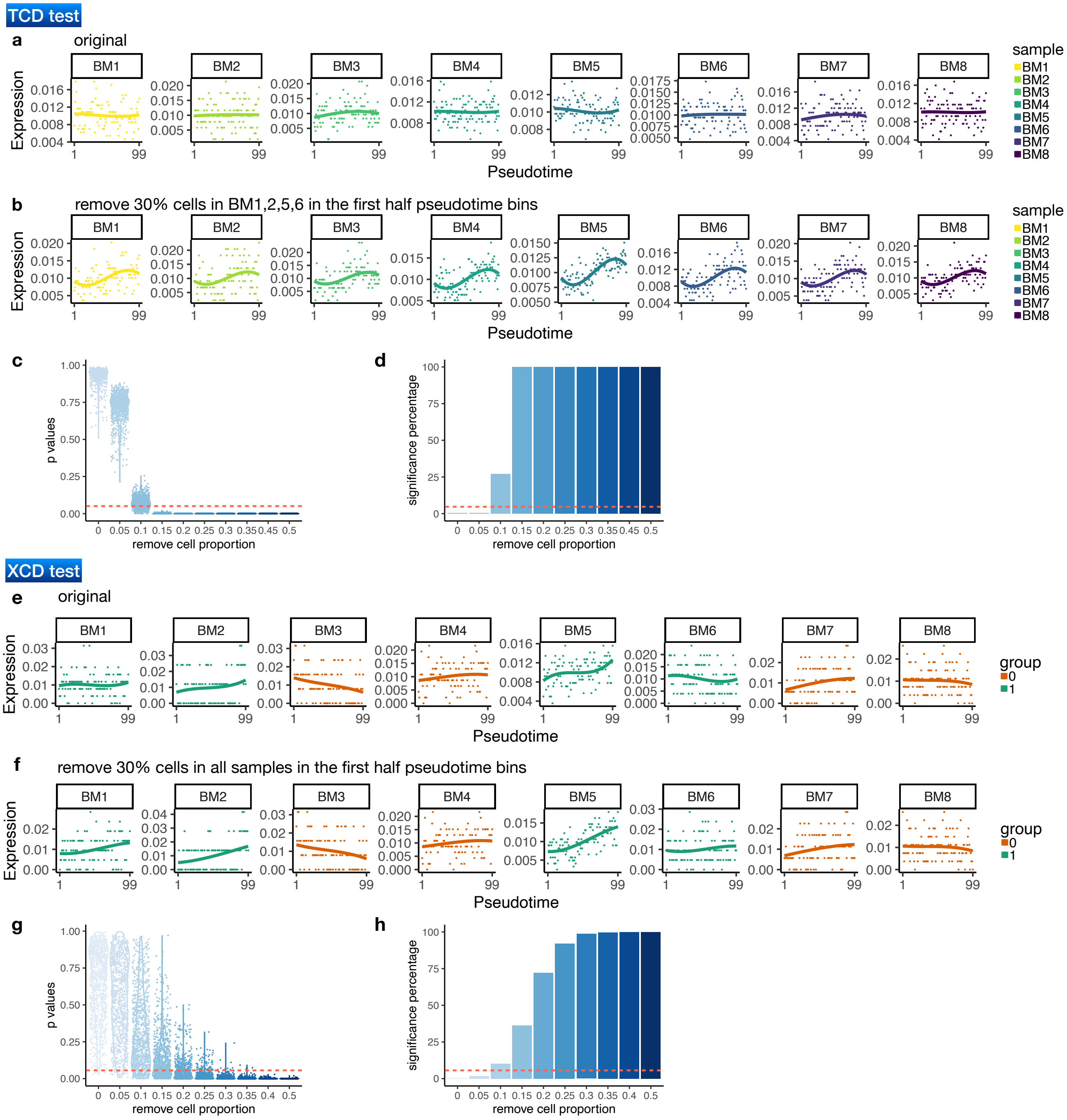

Figure S15. Simulation study for evaluating differential cell density tests. (a)-(d) TCD test simulation. **(a)** Null simulation setting of TCD test. Cell proportion in each of the 100 bins along pseudotime (denoted as dots) and the model-fitted curve for each HCA-BM sample are shown. The pseudotime of the cells have been permuted so that there is no time-dependent pattern. **(b)** Similar to (a) except that 50% of the cells in the first half pseudotime bins in each of the samples have been removed. **(c)** A violin plot showing the distribution of TCD test p-values (dots, one-sided) obtained from 1,000 simulations where a certain proportion (x-axis) of the cells in the first half pseudotime bins in each sample have been removed. **(d)** A bar plot showing the percentage of simulations (out of 1,000) that have reported a TCD test p-value < 0.05 (one-sided). **(e-h)** Similar to (a-d) except that it is in the XCD test simulation. Here, instead of removing cells in all samples, we only remove cells in the group 1 samples (BM1,2,5,6); and we do not permute the pseudotime. Source data are provided as a Source Data file.

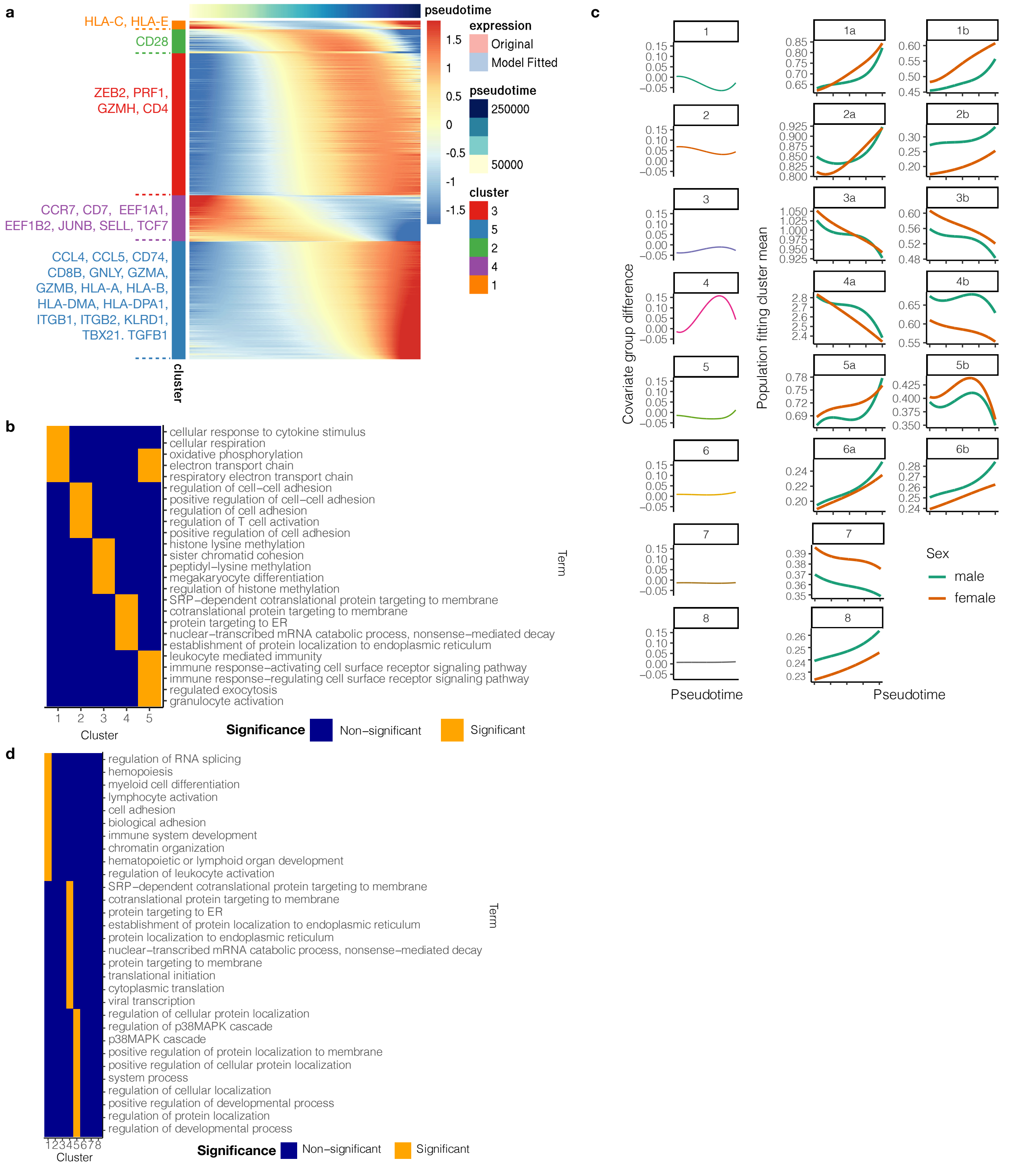

Figure S16. TDE and XDE genes and their enriched functions in TB data. (a) A heatmap showing the dynamic expression patterns of TDE genes along pseudotime. Each row represents a TDE gene. **(b)** Enriched GO terms of the TDE genes in each cluster shown in (a). **(c)** The model-fitted patterns of each XDE gene cluster are shown in the two columns on the right. Within each row, two clusters with the same trend difference (one with mean shift and one without) are labeled as a and b respectively. For example, Clusters 1a and 1b contain genes with the same trend difference pattern, but genes in 1b also have significant mean shift (i.e. meanSig). For each trend difference pattern, the difference between the male and female groups is shown in the column on the left. **(d)** Enriched GO terms of the XDE genes in each cluster shown in (c). GO analyses were conducted similarly to Fig. S4. Source data are provided as a Source Data file.

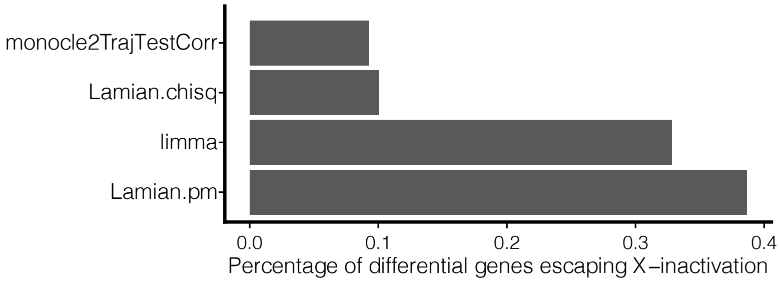

Figure S17. Comparison of Lamian and other methods based on the percentage of sex-associated XDE genes escaping X-chromosome inactivation (XCI) in TB data. XCI status is obtained from Balaton et al[2]. Source data are provided as a Source Data file.

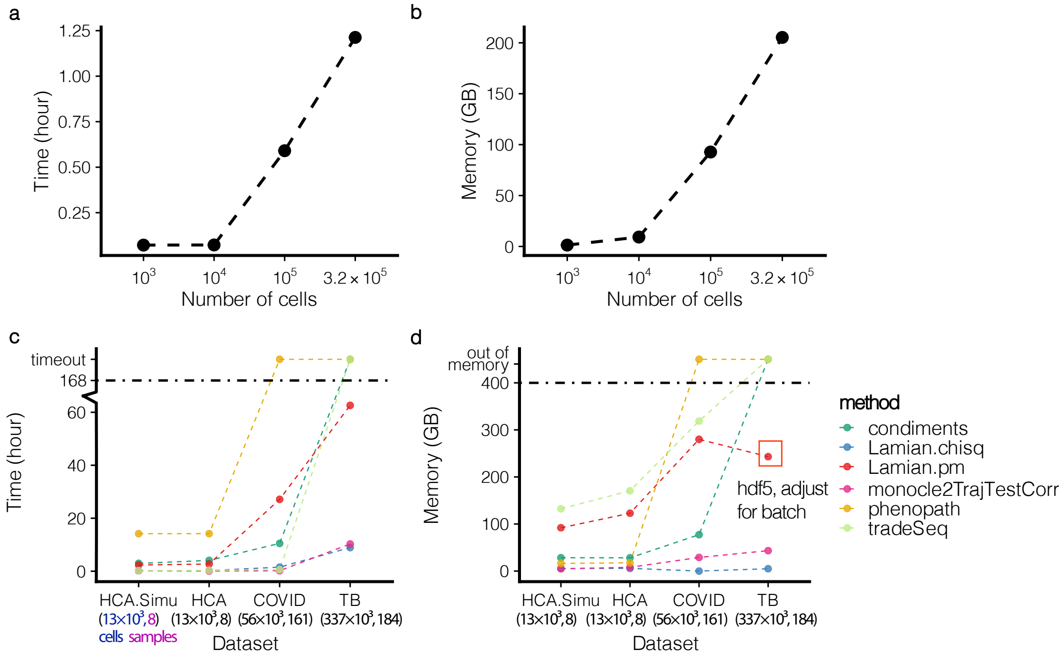

Figure S18. Comparison of computational time and memory usage of different methods. **(a)** Since the TB data analysis only involved Lamian modules 3 and 4, we further benchmarked the computational time required by modules 1 and 2. COVID-19 data with 161 samples were used to generate datasets with different numbers of cells. **(b)** Same as (a) but for memory usage in GB. **(c)** Computational time required for Lamian (both Lamian.chisq and Lamian.pm) and other XDE methods. Values more than one week (168 hours) are marked as *timeout*. **(d)** Same as (c) but for memory usage in GB. Values more than 400GB are marked as *out of memory*. Source data are provided as a Source Data file.

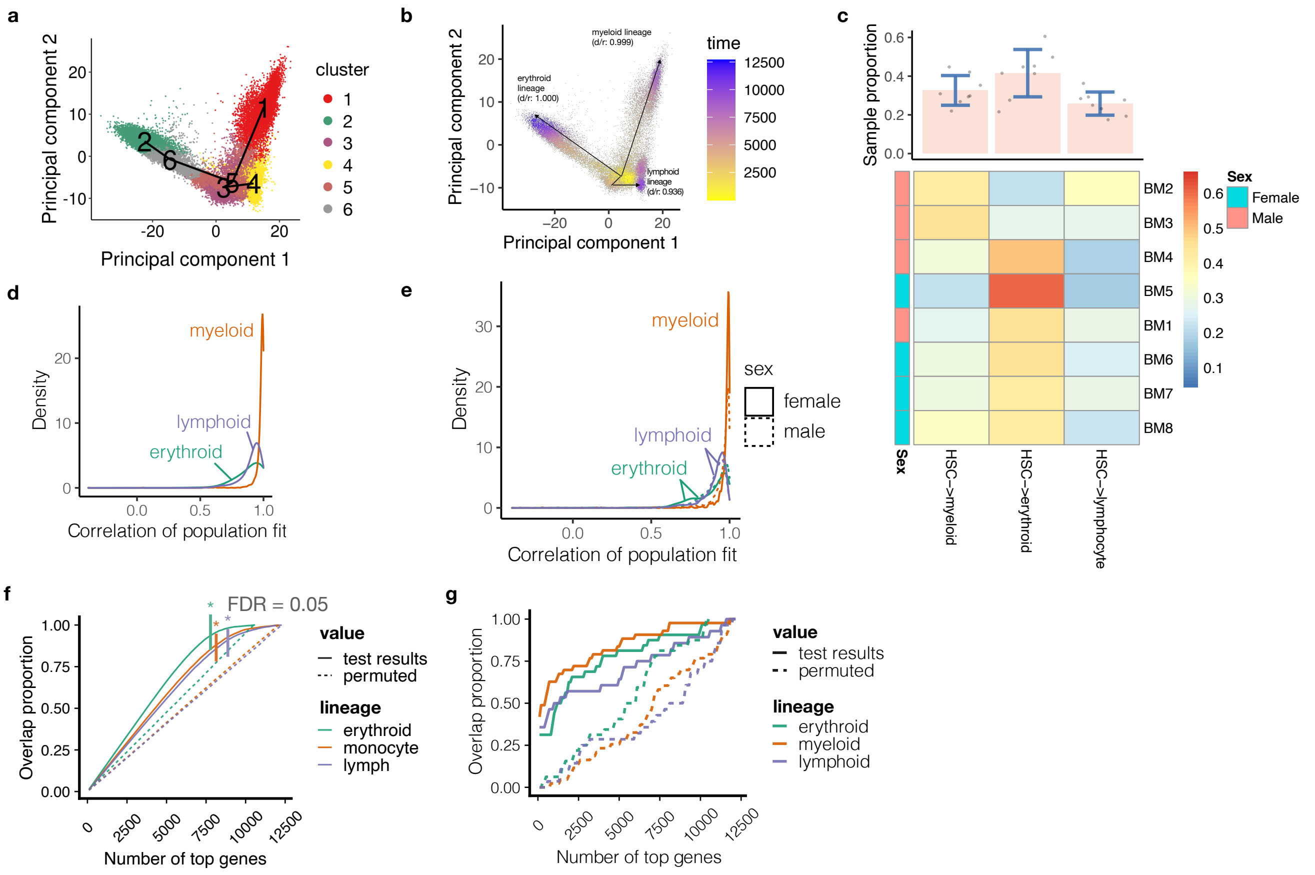

Figure S19. Lamian analysis of the HCA-BM data based on `Harmony` integration and comparison with `Seurat` integration. (a-b) Cells plotted with the top two latent variables from `Harmony`, colored by clusters (a) or pseudotime (b). Pseudotime was inferred using the top 10 latent variables output by `Harmony`. **(c)** Cell proportion on each lineage. The barplot shows the mean (pink bar) and mean ± SD (blue bars) of the branch cell proportion across $n = 8$ samples for each lineage. Cell proportions are displayed as dots and heatmap. **(d)** Distribution of the Pearson correlation coefficients between genes' pseudotemporal expression patterns fitted by `Seurat+Lamian` and those fitted by `Harmony+Lamian`. Pseudotemporal gene expression patterns were fitted as in TDE analysis. The correlation coefficient was computed for each gene using its fitted group-level curves. **(e)** Similar to (d) but the pseudotemporal curves are fitted as in XDE analysis. In other words, the correlation was analyzed within each sample group (female or male) separately. **(f)** The proportion of `Seurat+Lamian`-reported TDE genes (FDR<0.05) that can be found in the top $100n$ `Harmony+Lamian` TDE genes ($n = 1, 2, \ldots$) ("test results"). As a negative control, the gene list based on Harmony was also permuted to re-calculate the overlap ("permuted"). The vertical bars with "*" denote the FDR<0.05 cutoffs for `Harmony+Lamian` in the three lineages. **(g)** Similar to (f) but for XDE analysis. Source data are provided as a Source Data file.

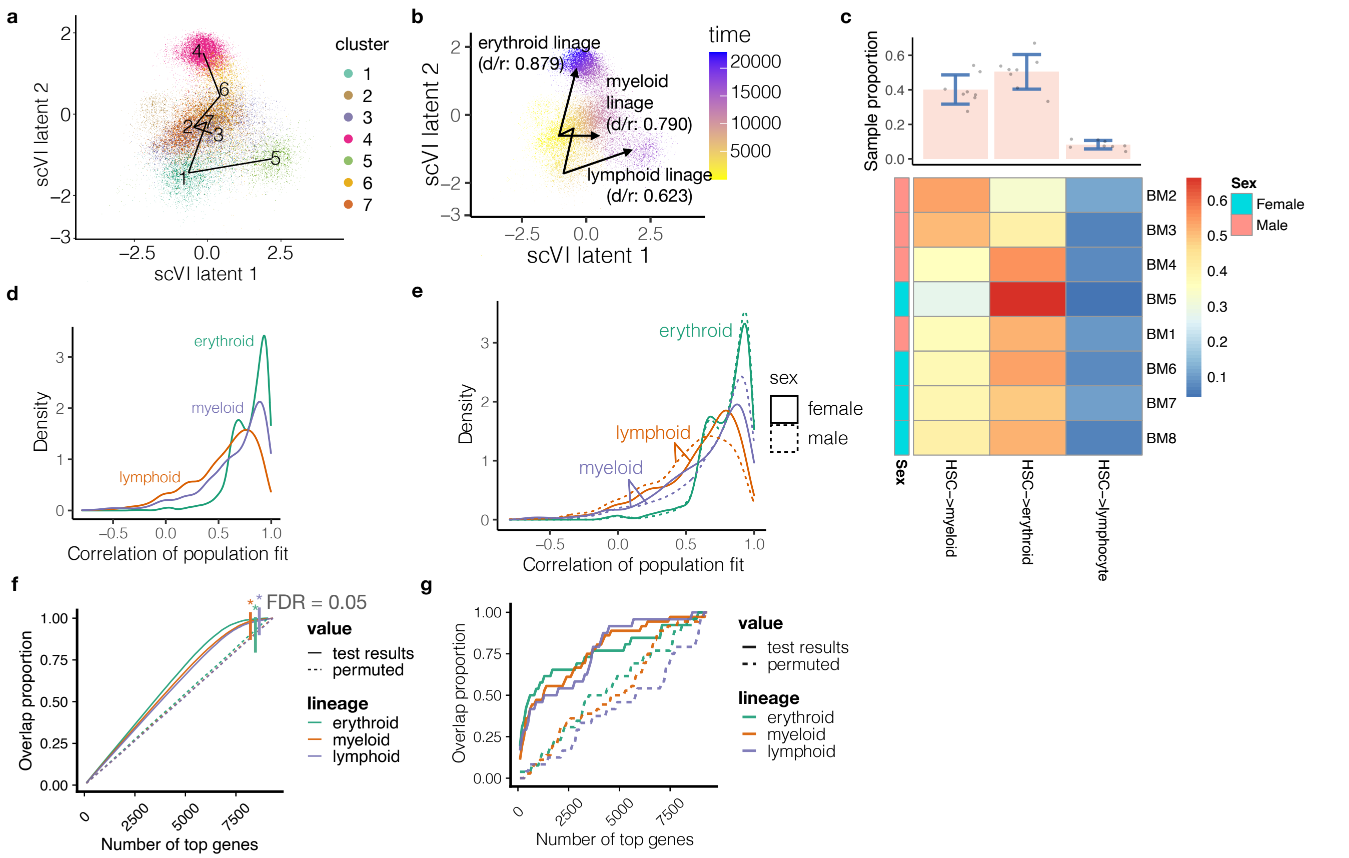

Figure S20. Lamian analysis of the HCA-BM data based on `scVI` integration and comparison with `Seurat` integration. (a-b) Cells plotted with top two latent variables from `scVI`, colored by clusters (a) or pseudotime (b). Pseudotime was inferred using the top 10 latent variables output by `scVI`. **(c)** Cell proportion on each lineage. The barplot shows the mean (pink bar) and mean $\pm$ SD (blue bars) of the branch cell proportion across $n = 8$ samples for each lineage. Cell proportions are displayed as dots and heatmap. **(d)** Distribution of the Pearson correlation coefficients between genes' pseudotemporal expression patterns fitted by `Seurat+Lamian` and those fitted by `scVI+Lamian`. Pseudotemporal gene expression patterns were fitted as in TDE analysis. The correlation coefficient was computed for each gene using its fitted group-level curves. **(e)** Similar to (d) but the pseudotemporal curves are fitted as in XDE analysis. In other words, the correlation was analyzed within each sample group (female or male) separately. **(f)** The proportion of `Seurat+Lamian`-reported TDE genes (FDR<0.05) that can be found in the top $100n$ `scVI+Lamian` TDE genes ($n = 1, 2, \ldots$) ("test results"). As a negative control, the gene list based on scVI was also permuted to re-calculate the overlap ("permuted"). The vertical bars with "*" denote the FDR<0.05 cutoffs for `scVI+Lamian` in the three lineages. **(g)** Similar to (f) but for XDE analysis. Source data are provided as a Source Data file.

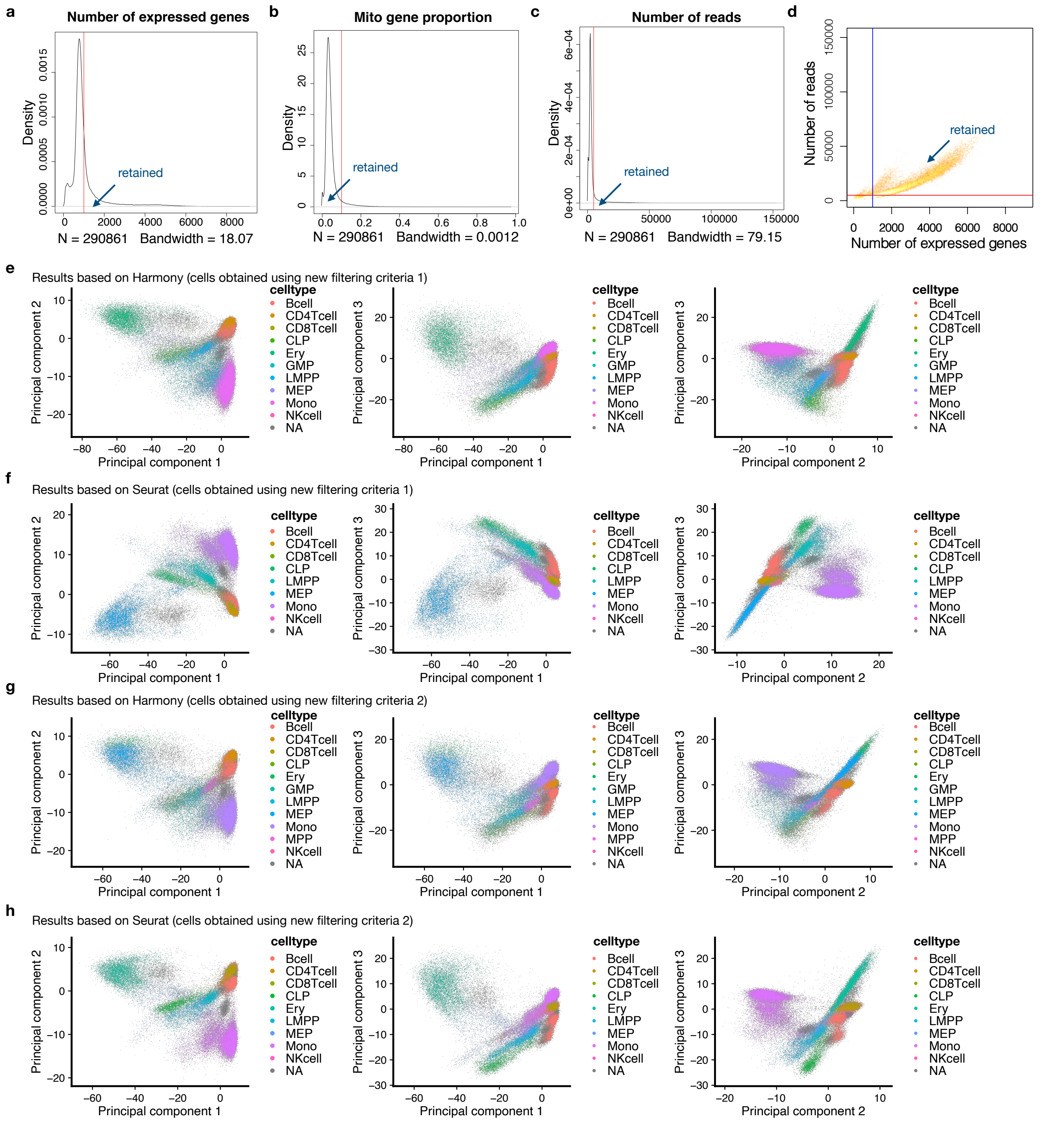

Figure S21. Quality control (QC) plots and the integration results from `Harmony` and `Seurat` with relaxed quality filters. (a)-(d) Current filtering criteria. **(e)-(f)** Integration results from `Harmony` (e) and `Seurat` RPCA (f) after relaxing the number of expressed genes per cell cutoff from 1000 to 500. In Harmony (e), 6/27 clusters are unidentifiable cell types (NA) which occupy 10831 out of 255547 cells. In `Seurat` (f), 7/28 clusters are unidentifiable cell types (NA) which occupy 11409 out of 255547 cells. **(g)-(h)** Integration results from `Harmony` (g) and `Seurat` RPCA (h) after changing the number of expressed genes cutoff from 1000 to the median number of expressed genes. In `Harmony` (g), 9 out of 29 clusters are NA, i.e. 11995 out of 164917 cells. In `Seurat` (h), 8 out of 27 clusters are NA, i.e. 9673 out of 164917 cells. Source data are provided as a Source Data file.

Supplementary Notes

1 Model

1.1 EM algorithm for fitting the Lamian model

This section presents the Expectation-Maximization(EM) algorithm used to fit the Lamian model for a given gene to estimate the unknown parameters $\Theta = \{\beta, \Omega, \alpha, \eta\}$ and infer σ_s^2 based on the gene's observed data. Here $\sigma_s^2, \alpha, \eta \in \mathbb{R}$, $\Omega \in \mathbb{R}^{(K+1)\times(K+1)}$, $\beta \in \mathbb{R}^{(K+1)(V+1)}$. In the EM algorithm, we treat sample-level random effects $\mathbf{u} \equiv \{\mathbf{u}_1, \ldots, \mathbf{u}_S\}$ and their variance $\sigma^2 \equiv \{\sigma_1^2, \ldots, \sigma_S^2\}$ as missing data. The observed data are $\mathbf{y} \equiv \{\mathbf{y}_1, \ldots, \mathbf{y}_S\}$. The complete data are $\{\mathbf{y}, \mathbf{u}, \sigma^2\}$.

For a given gene, the complete data likelihood is

$$
\begin{aligned}
L(\Theta) =& P(\mathbf{y}, \mathbf{u}, \sigma^2|\Theta) \\
=& \prod_{s=1}^{S} \left[P(\mathbf{y}_s|\mathbf{u}_s, \beta, \sigma_s^2) P(\mathbf{u}_s|\sigma_s^2, \Omega) P(\sigma_s^2|\alpha, \eta) \right] \\
=& \prod_s \left[(2\pi\sigma_s^2)^{-C_s/2} \exp\left\{ -\frac{1}{2\sigma_s^2} [\mathbf{y}_s - \Phi_s \cdot (\mathbf{X}_s\beta + \mathbf{u}_s)]^T [\mathbf{y}_s - \Phi_s \cdot (\mathbf{X}_s\beta + \mathbf{u}_s)] \right\} \cdot \right. \\
& \left. (2\pi)^{-(K+1)/2} |\sigma_s^2 \Omega|^{-1/2} \exp\left\{ -\frac{1}{2} \mathbf{u}_s^T (\sigma_s^2 \Omega)^{-1} \mathbf{u}_s \right\} \frac{\eta^\alpha}{\Gamma(\alpha)} (\sigma_s^2)^{-\alpha-1} \exp(-\frac{\eta}{\sigma_s^2}) \right] \\
=& \prod_s \left[(2\pi)^{-(C_s+K+1)/2} (\sigma_s^2)^{-(C_s+K+1)/2-\alpha-1} |\Omega|^{-1/2} \frac{\eta^\alpha}{\Gamma(\alpha)} \cdot \right. \\
& \left. \exp\left\{ -\frac{1}{2\sigma_s^2} [(\mathbf{y}_s - \Phi_s\mathbf{X}_s\beta)^T (\mathbf{y}_s - \Phi_s\mathbf{X}_s\beta) - 2(\mathbf{y}_s - \Phi_s\mathbf{X}_s\beta)^T \Phi_s \mathbf{u}_s + \mathbf{u}_s^T (\Phi_s^T \Phi_s + \Omega^{-1}) \mathbf{u}_s + 2\eta] \right\} \right] \\
=& \prod_s \left[(2\pi)^{-(C_s+K+1)/2} (\sigma_s^2)^{-(C_s+K+1)/2-\alpha-1} |\Omega|^{-1/2} \frac{\eta^\alpha}{\Gamma(\alpha)} \cdot \exp\left\{ -\frac{1}{2\sigma_s^2} [\mathbf{L}_s - 2\mathbf{K}_s\mathbf{u}_s + \mathbf{u}_s^T \mathbf{J}_s \mathbf{u}_s + 2\eta] \right\} \right]
\end{aligned}
$$

where

$$
\begin{aligned}
\mathbf{L}_s &= (\mathbf{y}_s - \Phi_s\mathbf{X}_s\beta)^T (\mathbf{y}_s - \Phi_s\mathbf{X}_s\beta) \\
\mathbf{K}_s &= (\mathbf{y}_s - \Phi_s\mathbf{X}_s\beta)^T \Phi_s \\
\mathbf{J}_s &= \Phi_s^T \Phi_s + \Omega^{-1}
\end{aligned}
$$

The complete data log-likelihood is

$$
\begin{aligned}
l(\Theta) = \sum_{s=1}^{S} & \left[(-\frac{C_s+K+1}{2} - \alpha - 1) \log(\sigma_s^2) - \frac{1}{2} \log(|\Omega|) + \alpha \log(\eta) - \log(\Gamma(\alpha)) \right. \\
& \left. - \frac{1}{2\sigma_s^2} [\mathbf{L}_s - 2\mathbf{K}_s\mathbf{u}_s + \mathbf{u}_s^T \mathbf{J}_s \mathbf{u}_s + 2\eta] \right] + constant \qquad (1)
\end{aligned}
$$

Here the *constant* term does not involve the unknown parameters.

The EM algorithm iterates between the E-step and the M-step below until convergence.

1.1.1 E-step

In iteration $t+1$, the E-step computes the Q-function $Q(\Theta|\Theta^{(t)})$, which is the expectation of the complete data log-likelihood $l(\Theta)$ with respect to the conditional distribution of missing data $\mathbf{u}$ and σ^2 given the observed data $\mathbf{y}$ and old parameter values $\Theta^{(t)}$. Based on Equation 1, this involves evaluating (1) $E_{\sigma_s^2|\mathbf{y}_s,\Theta^{(t)}}[\log(\sigma_s^2)]$, (2) $E_{\mathbf{u}_s,\sigma_s^2|\mathbf{y}_s,\Theta^{(t)}}[\frac{\mathbf{u}_s}{\sigma_s^2}]$, and (3) $E_{\mathbf{u}_s,\sigma_s^2|\mathbf{y}_s,\Theta^{(t)}}[\frac{\mathbf{u}_s^T\mathbf{J}_s\mathbf{u}_s}{\sigma_s^2}]$.

Note that

$$
\begin{aligned}
P(\mathbf{u}_s, \sigma_s^2|\mathbf{y}_s, \Theta) \propto& P(\mathbf{y}_s|\mathbf{u}_s, \sigma_s^2, \Theta) P(\mathbf{u}_s|\sigma_s^2, \Theta) P(\sigma_s^2|\Theta) \\
\propto& (\sigma_s^2)^{-(C_s+K+1)/2-\alpha-1} \exp\left\{ -\frac{1}{2\sigma_s^2} [\mathbf{L}_s - 2\mathbf{K}_s\mathbf{u}_s + \mathbf{u}_s^T \mathbf{J}_s \mathbf{u}_s + 2\eta] \right\} \\
=& (\sigma_s^2)^{-(C_s+K+1)/2-\alpha-1} \exp\left\{ -\frac{1}{2\sigma_s^2} [\mathbf{L}_s - \mathbf{K}_s\mathbf{J}_s^{-1}\mathbf{K}_s^T + (\mathbf{u}_s - \mathbf{J}_s^{-1}\mathbf{K}_s^T)^T \mathbf{J}_s (\mathbf{u}_s - \mathbf{J}_s^{-1}\mathbf{K}_s^T) + 2\eta] \right\}
\end{aligned}
$$

$$=(\sigma_s^2)^{-(C_s+K+1)/2-\alpha-1}\exp\left\{-\frac{1}{2\sigma_s^2}[\mathbf{L}_s-\mathbf{K}_s\mathbf{J}_s^{-1}\mathbf{K}_s^T+2\eta]\right\}\cdot$$
$$\exp\left\{-\frac{1}{2\sigma_s^2}[(\mathbf{u}_s-\mathbf{J}_s^{-1}\mathbf{K}_s^T)^T\mathbf{J}_s(\mathbf{u}_s-\mathbf{J}_s^{-1}\mathbf{K}_s^T)]\right\}$$

Thus,

$$\mathbf{u}_s|\sigma_s^2,\mathbf{y}_s,\Theta\sim N(\mathbf{J}_s^{-1}\mathbf{K}_s^T,\sigma_s^2\mathbf{J}_s^{-1}) \tag{2}$$
$$\sigma_s^2|\mathbf{y}_s,\Theta\sim IG(\alpha+C_s/2,\eta+(\mathbf{L}_s-\mathbf{K}_s\mathbf{J}_s^{-1}\mathbf{K}_s^T)/2) \tag{3}$$

Here the inverse-Gamma distribution for $\sigma_s^2|\mathbf{y}_s,\Theta$ is derived based on

$$P(\sigma_s^2|\mathbf{y}_s,\Theta)=\int P(\mathbf{u}_s,\sigma_s^2|\mathbf{y}_s,\Theta)d\mathbf{u}_s$$
$$\propto(\sigma_s^2)^{-(C_s+K+1)/2-\alpha-1}\exp\left\{-\frac{1}{2\sigma_s^2}[\mathbf{L}_s-\mathbf{K}_s\mathbf{J}_s^{-1}\mathbf{K}_s^T+2\eta]\right\}|\mathbf{J}_s^{-1}\sigma_s^2|^{1/2}$$
$$\propto(\sigma_s^2)^{-C_s/2-\alpha-1}\exp\left\{-\frac{1}{2\sigma_s^2}[\mathbf{L}_s-\mathbf{K}_s\mathbf{J}_s^{-1}\mathbf{K}_s^T+2\eta]\right\}$$

Based on the properties of inverse-Gamma distribution, we have

$$A_s^{(t)}\equiv E_{\sigma_s^2|\mathbf{y}_s,\Theta^{(t)}}[\log(\sigma_s^2)]=\log(\eta^{(t)}+(\mathbf{L}_s^{(t)}-\mathbf{K}_s^{(t)}(\mathbf{J}_s^{(t)})^{-1}(\mathbf{K}_s^{(t)})^T)/2)-\psi(\alpha^{(t)}+C_s/2) \tag{4}$$

where $\psi(\cdot)$ is digamma function.

Let

$$N_s^{(t)}\equiv E_{\sigma_s^2|\mathbf{y}_s,\Theta^{(t)}}[\frac{1}{\sigma_s^2}]=\frac{2\alpha^{(t)}+C_s}{2\eta^{(t)}+\mathbf{L}_s^{(t)}-\mathbf{K}_s^{(t)}(\mathbf{J}_s^{(t)})^{-1}(\mathbf{K}_s^{(t)})^T} \tag{5}$$

Since $\mathbf{u}_s|\sigma_s^2,\mathbf{y}_s,\Theta\sim N(\mathbf{J}_s^{-1}\mathbf{K}_s^T,\sigma_s^2\mathbf{J}_s^{-1})$, we have

$$E_{\mathbf{u}_s,\sigma_s^2|\mathbf{y}_s,\Theta^{(t)}}[\frac{\mathbf{u}_s}{\sigma_s^2}]=E_{\sigma_s^2|\mathbf{y}_s,\Theta^{(t)}}\left[E_{\mathbf{u}_s|\sigma_s^2,\mathbf{y}_s,\Theta^{(t)}}[\frac{\mathbf{u}_s}{\sigma_s^2}]\right]$$
$$=E_{\sigma_s^2|\mathbf{y}_s,\Theta^{(t)}}\left[\frac{(\mathbf{J}_s^{(t)})^{-1}(\mathbf{K}_s^{(t)})^T}{\sigma_s^2}\right]$$
$$=N_s^{(t)}(\mathbf{J}_s^{(t)})^{-1}(\mathbf{K}_s^{(t)})^T \tag{6}$$

and

$$E_{\mathbf{u}_s,\sigma_s^2|\mathbf{y}_s,\Theta^{(t)}}[\frac{\mathbf{u}_s^T\mathbf{J}_s\mathbf{u}_s}{\sigma_s^2}]=E_{\sigma_s^2|\mathbf{y}_s,\Theta^{(t)}}\left[E_{\mathbf{u}_s|\sigma_s^2,\mathbf{y}_s,\Theta^{(t)}}[\frac{\mathbf{u}_s^T\mathbf{J}_s\mathbf{u}_s}{\sigma_s^2}]\right]$$
$$=E_{\sigma_s^2|\mathbf{y}_s,\Theta^{(t)}}\left[\frac{1}{\sigma_s^2}\mathbf{K}_s^{(t)}(\mathbf{J}_s^{(t)})^{-1}\mathbf{J}_s(\mathbf{J}_s^{(t)})^{-1}(\mathbf{K}_s^{(t)})^T\right]+tr(\mathbf{J}_s(\mathbf{J}_s^{(t)})^{-1})$$
$$=\mathbf{K}_s^{(t)}(\mathbf{J}_s^{(t)})^{-1}\mathbf{J}_s(\mathbf{J}_s^{(t)})^{-1}(\mathbf{K}_s^{(t)})^TE_{\sigma_s^2|\mathbf{y}_s,\Theta^{(t)}}[\frac{1}{\sigma_s^2}]+tr(\mathbf{J}_s(\mathbf{J}_s^{(t)})^{-1})$$
$$=N_s^{(t)}\mathbf{K}_s^{(t)}(\mathbf{J}_s^{(t)})^{-1}\mathbf{J}_s(\mathbf{J}_s^{(t)})^{-1}(\mathbf{K}_s^{(t)})^T+tr(\mathbf{J}_s(\mathbf{J}_s^{(t)})^{-1}) \tag{7}$$

Based on Equations 4-7, the Q-function is

$$Q(\Theta|\Theta^{(t)})=E_{\mathbf{u},\sigma^2|\mathbf{y},\Theta^{(t)}}l(\Theta)$$
$$=\sum_s\left[-\alpha A_s^{(t)}-\frac{1}{2}\log(|\Omega|)+\alpha\log(\eta)-\log(\Gamma(\alpha))-\right.$$

$$
\begin{aligned}
&E_{\mathbf{u}_s,\sigma_s^2|\mathbf{y}_s,\Theta^{(t)}}\left(\frac{1}{2\sigma_s^2}[\mathbf{L}_s - 2\mathbf{K}_s\mathbf{u}_s + \mathbf{u}_s^T\mathbf{J}_s\mathbf{u}_s + 2\eta]\right)\Bigg] + constant \\
=&\sum_s \Bigg[-\alpha A_s^{(t)} - \frac{1}{2}\log(|\Omega|) + \alpha\log(\eta) - \log(\Gamma(\alpha)) - \frac{1}{2}N_s^{(t)}\mathbf{L}_s + \mathbf{K}_s N_s^{(t)}(\mathbf{J}_s^{(t)})^{-1}(\mathbf{K}_s^{(t)})^T \\
&-\frac{1}{2}N_s^{(t)}\mathbf{K}_s^{(t)}(\mathbf{J}_s^{(t)})^{-1}\mathbf{J}_s(\mathbf{J}_s^{(t)})^{-1}(\mathbf{K}_s^{(t)})^T - \frac{1}{2}tr(\mathbf{J}_s(\mathbf{J}_s^{(t)})^{-1}) - N_s^{(t)}\eta\Bigg] + constant \\
=&\sum_s \Bigg[-\alpha A_s^{(t)} - \frac{1}{2}\log(|\Omega|) + \alpha\log(\eta) - \log(\Gamma(\alpha)) \\
&+ N_s^{(t)}\left(-\frac{1}{2}(\Phi_s\mathbf{X}_s\beta)^T(\Phi_s\mathbf{X}_s\beta) + (\Phi_s\mathbf{X}_s\beta)^T\mathbf{y}_s - (\Phi_s\mathbf{X}_s\beta)^T\Phi_s(\mathbf{J}_s^{(t)})^{-1}(\mathbf{K}_s^{(t)})^T - \eta\right) \\
&-\frac{1}{2}\left(tr(\Omega^{-1}(\mathbf{J}_s^{(t)})^{-1}) + N_s^{(t)}\mathbf{K}_s^{(t)}(\mathbf{J}_s^{(t)})^{-1}\Omega^{-1}(\mathbf{J}_s^{(t)})^{-1}(\mathbf{K}_s^{(t)})^T\right)\Bigg] + constant
\end{aligned} \tag{8}
$$

1.1.2 M-step

By maximizing the Q-function with respect to the unknown parameters Θ, we obtain the new parameter estimates.

- $\eta^{(t+1)}$: it is the solution to

$$
\log\eta = \sum_s A_s^{(t)}/S + \psi(\eta\sum_s N_s^{(t)}/S) \tag{9}
$$

  where $\psi(\cdot)$ is the digamma function. This can be solved using bound constrained optimization[3].

- $\alpha^{(t+1)}$:

$$
\alpha^{(t+1)} = \eta^{(t+1)}\sum_s N_s^{(t)}/S \tag{10}
$$

- $\beta^{(t+1)}$:

$$
\beta^{(t+1)} = \left(\sum_s N_s^{(t)}(\Phi_s\mathbf{X}_s)^T(\Phi_s\mathbf{X}_s)\right)^{-1}\left(\sum_s N_s^{(t)}\left[(\Phi_s\mathbf{X}_s)^T(\mathbf{y}_s - \Phi_s(\mathbf{J}_s^{(t)})^{-1}(\mathbf{K}_s^{(t)})^T)\right]\right) \tag{11}
$$

- $\Omega^{(t+1)}$:

$$
\Omega^{(t+1)} = \sum_s\left[(\mathbf{J}_s^{(t)})^{-1} + N_s^{(t)}(\mathbf{J}_s^{(t)})^{-1}(\mathbf{K}_s^{(t)})^T(\mathbf{K}_s^{(t)})(\mathbf{J}_s^{(t)})^{-1}\right]/S \tag{12}
$$

1.2 Observed data likelihood

To compare two models, the likelihood ratio statistic is computed based on the observed data likelihood. The observed data likelihood for a Lamian model is:

$$
L_{obs}(\Theta) = P(\mathbf{y}|\Theta) = \prod_s P(\mathbf{y}_s|\Theta) \tag{13}
$$

Here

$$
\begin{aligned}
P(\mathbf{y}_s|\Theta) &= \int\int P(\mathbf{y}_s, \mathbf{u}_s, \sigma_s^2|\Theta)\,d\mathbf{u}_s\,d\sigma_s^2 \\
&= \int\int P(\mathbf{y}_s|\mathbf{u}_s,\beta,\sigma_s^2)P(\mathbf{u}_s|\sigma_s^2,\Omega)P(\sigma_s^2|\alpha,\eta)\,d\mathbf{u}_s\,d\sigma_s^2 \\
&= \int\int\Bigg[(2\pi)^{-(C_s+K+1)/2}(\sigma_s^2)^{-(C_s+K+1)/2-\alpha-1}|\Omega|^{-1/2}\frac{(\eta)^\alpha}{\Gamma(\alpha)} \\
&\quad \exp\left\{-\frac{1}{2\sigma_s^2}[\mathbf{L}_s - 2\mathbf{K}_s\mathbf{u}_s + \mathbf{u}_s^T\mathbf{J}_s\mathbf{u}_s + 2\eta]\right\}\Bigg]\,d\sigma_s^2\,d\mathbf{u}_s
\end{aligned}
$$

$$=(2\pi)^{-(C_s+K+1)/2}|\Omega|^{-1/2}\frac{(\eta)^\alpha}{\Gamma(\alpha)}\Gamma\left(\frac{C_s+K+1}{2}+\alpha\right)$$
$$\int\left(\frac{1}{2}[\mathbf{L}_s-2\mathbf{K}_s\mathbf{u}_s+\mathbf{u}_s^T\mathbf{J}_s\mathbf{u}_s+2\eta]\right)^{-(C_s+K+1)/2-\alpha}d\mathbf{u}_s$$
$$=(2\pi)^{-(C_s+K+1)/2}|\Omega|^{-1/2}\frac{(\eta)^\alpha}{\Gamma(\alpha)}\Gamma\left(\frac{C_s+K+1}{2}+\alpha\right)$$
$$\int\left(\frac{1}{2}[\mathbf{L}_s+2\eta+(\mathbf{u}_s-\mathbf{J}_s^{-1}\mathbf{K}_s^T)^T\mathbf{J}_s(\mathbf{u}_s-\mathbf{J}_s^{-1}\mathbf{K}_s^T)-\mathbf{K}_s\mathbf{J}_s^{-1}\mathbf{K}_s^T]\right)^{-(C_s+K+1)/2-\alpha}d\mathbf{u}_s$$
$$=(2\pi)^{-(C_s+K+1)/2}|\Omega|^{-1/2}\frac{(\eta)^\alpha}{\Gamma(\alpha)}\Gamma\left(\frac{C_s+K+1}{2}+\alpha\right)2^{(C_s+K+1)/2+\alpha}$$
$$\int\left(\mathbf{L}_s+2\eta-\mathbf{K}_s\mathbf{J}_s^{-1}\mathbf{K}_s^T+(\mathbf{u}_s-\mathbf{J}_s^{-1}\mathbf{K}_s^T)^T\mathbf{J}_s(\mathbf{u}_s-\mathbf{J}_s^{-1}\mathbf{K}_s^T)\right)^{-(C_s+K+1)/2-\alpha}d\mathbf{u}_s$$

(Note the connection between the integrand and the probability density function of multivariate t-distribution)

$$=(2\pi)^{-(C_s+K+1)/2}|\Omega|^{-1/2}\frac{(\eta)^\alpha}{\Gamma(\alpha)}\Gamma\left(\frac{C_s+K+1}{2}+\alpha\right)2^{(C_s+K+1)/2+\alpha}$$
$$(\mathbf{L}_s+2\eta-\mathbf{K}_s\mathbf{J}_s^{-1}\mathbf{K}_s^T)^{-(C_s+K+1)/2-\alpha}\frac{\Gamma\left(\frac{C_s+2\alpha}{2}\right)(C_s+2\alpha)^{(K+1)/2}\pi^{(K+1)/2}}{\Gamma\left(\frac{C_s+K+1}{2}+\alpha\right)}$$
$$\left|\frac{(\mathbf{L}_s+2\eta-\mathbf{K}_s\mathbf{J}_s^{-1}\mathbf{K}_s^T)(\mathbf{J}_s)^{-1}}{C_S+2\alpha}\right|^{1/2}$$
$$=\frac{(2\eta)^\alpha\Gamma(C_s/2+\alpha)}{(\pi)^{C_s/2}\Gamma(\alpha)|\Omega|^{1/2}|\mathbf{J}_s|^{1/2}(\mathbf{L}_s+2\eta-\mathbf{K}_s\mathbf{J}_s^{-1}\mathbf{K}_s^T)^{C_s/2+\alpha}}$$

Thus, the log of the observed data likelihood used to construct the likelihood ratio test statistic is

$$l_{obs}(\Theta)=\sum_s\log P(\mathbf{y}_s|\Theta)$$
$$=\sum_s\left[\alpha\log(2\eta)+\log\Gamma\left(\frac{C_s}{2}+\alpha\right)-\frac{C_s}{2}\log(\pi)-\log\Gamma(\alpha)\right.$$
$$\left.-\frac{1}{2}\log(|\Omega|)-\frac{1}{2}\log(|\mathbf{J}_s|)-\left(\frac{C_s}{2}+\alpha\right)\log(\mathbf{L}_s+2\eta-\mathbf{K}_s\mathbf{J}_s^{-1}\mathbf{K}_s^T)\right] \quad (14)$$

where the parameters Θ are set to be the maximum likelihood estimates (MLE) obtained from the EM algorithm (i.e., $\Theta^{(t)}$ from the last EM iteration).

2 Additional notes for filtering cells in HCA-BM data

For this dataset, we retained cells with $\geq$ 1000 expressed genes, $\leq$ 10% mitochondrial reads, and $\geq$ 5000 reads (Fig.S21a-c). The 10% mitochondrial reads cutoff is not a strong filter and retains most of the cells. The number of expressed genes cutoff was set as 1000, which is a strong filter and removes the majority of cells. However, applying less stringent cutoffs resulted in noisy low dimensional representation of cells that did not clearly capture the known biology. For example, setting the number of expressed gene cutoff to 500 allowed us to retain 255,547 cells. However, the three cell differentiation lineages were unclear in both `Seurat`- and `Harmony`-integrated results. Moreover, both methods were not able to identify any cluster annotated as hematopoietic stem cell (HSC), and a number of clusters were annotated as NA (unidentifiable cell types, colored by grey) (Fig. S21 e,f). These indicate that for this well-studied system, setting the cutoff to be 500 was not a good choice. Similarly, we also tried to set the cutoff to be the median number of expressed genes across cells. The results were also noisy and did not clearly capture the three lineages (Fig.S21 g,h). Since HCA-BM data serves as an example dataset for method illustration, we decided to apply the stringent cutoff 1000 so that we can capture the known biology and hence use the known biology to benchmark downstream differential expression analysis. For the read count cutoff, the number of reads and the number of expressed genes are highly correlated (Fig.S21d). We choose 5000 as the reads cutoff simply because most cells with $\geq$1000 expressed genes also have $\geq$5000 reads. In other words, 5000 reads cutoff is not a strong filter on top of the number of expressed gene filter and does not influence the filtering results much.

3 Evaluation

3.1 Evaluation of TDE detection

We compared Lamian with several state-of-the-art TDE detection methods including Monocle2/3, tradeSeq (which is the TDE method used by Slingshot), and TSCAN. We also tested `pseudotimeDE`, but it failed to run on our test data within one week.

We first created a null simulation (TDE simulation 1) using the bone marrow data by permuting cells' pseudotime within each sample. This creates a dataset where no genes are differential along pseudotime but sample-level variation is retained (Fig. S14a). Instead of considering the variability across samples, existing methods analyze all cells as if they were from a single sample. Therefore, they all reported a large number of false positives at the claimed 5% FDR cutoff. By contrast, Lamian.pm successfully controlled the FDR and did not report any false positive (Fig. S14a,b).

In another analysis (TDE simulation 2) which builds upon the null simulation above, we added spike-in signals *in silico* with varying signal-to-noise ratio to a random set of genes which provide gold standard TDE genes. In other words, after creating the above null simulation data which contain no TDE genes, we randomly selected 20% (1814) genes to spike in signals to create the gold standard TDE genes (gs genes). To create the spike-in signals, we first selected source genes in the same way as in the XDE simulation. Next, the source genes were categorized into four groups from the weakest signal strength (group 1) to the highest signal strength (group 4), using a similar procedure to that in the spike-in simulation in XDE detection, except that all cells were used here instead of only cells in sample group 0. In each sample, we added the gene expression profiles of the source genes in the same strength group to those of the gold standard TDE genes. The signal spike-in step was operated in SAVER-imputed values and counts in parallel. This results in a dataset where we know which genes are TDE. At the same time, the sample-level variability in real data is also retained. The spike-in signals were simulated from 5 different patterns, which were successfully recovered by Lamian via unsupervised clustering of TDE genes (Fig. S14c-e). Compared with other TDE detection methods, Lamian.pm not only offered the highest sensitivity to detect TDE genes but also controlled FDR (i.e. True FDR - Reported FDR < 0). Lamian.chisq offered comparable power but slightly underestimated FDR. By contrast, the other methods either failed to control FDR or had lower power as measured by the area under the sensitivity vs. true FDR curve (AUC) (Fig. S14f).

3.2 Evaluation of TCD detection

To evaluate TCD detection, we first created a null dataset by randomly permuting the pseudotime of the cells in erythroid lineage within each sample of the HCA bone marrow dataset. After permutation, there is no temporal variation expected (Fig. S15a). Next, we divided the pseudotime into 100 non-overlapping bins. To add spike-in signals, we randomly excluded $x\%$ ($x = 0, 5, 10, \ldots, 50$) cells from each of the samples in the first half of the pseudotime bins. This results in cell density changes along pseudotime where the cell density in the first half of the bins is expected to drop (Fig. S15b). Increasing x results in a larger drop. For each x value, we repeated the simulation 1000 times and applied TCD test to each simulation dataset, resulting in 1000 p-values. The distribution of the p-values for each x is shown in Fig. S15c. The p-vallues became smaller when excluding more cells (larger x) (Fig. S15c). Fig. S15d shows the percentage of p-vallues that were smaller than the α=0.05 significance cutoff. When no cells were excluded ($x = 0$), one does not expect any cell density change along pseudotime. Indeed, we observed that less than 5% p-vallues from TCD tests were below 0.05, indicating that TCD test correctly controlled the Type I error rate. When cells were excluded with increasing proportion (i.e., increasing x), the percentage of p-vallues that were below 0.05 also increased, indicating an increasing power of the TCD test for detecting increasingly larger cell density changes along pseudotime (Fig. S15d).

3.3 Evaluation of XCD detection

To evaluate XCD detection, we begin with creating a null dataset. To do so, the eight HCA bone marrow samples were randomly partitioned into two groups (BM1,2,5,6 as group 1, and the remaining samples as group 0). We divided the pseudotime in erythroid lineage into 100 non-overlapping bins. Within each bin and for each sample, we calculated the proportion of cells falling into that bin (Fig. S15e). For each bin, the median of cell proportions within each sample group was calculated. For the sample group with a larger median cell proportion, we randomly excluded cells from that group so that the two sample groups had the same median cell proportion after exclusion. This results in a null dataset without cell density differences between the two sample groups (Fig. S15e). We then randomly bootstrapped the cells and created 1000 null datasets.

To add spike-in differential signals to null data, we randomly excluded $x\%$ ($x = 0, 5, 10, \ldots, 50$) cells from each sample in one sample group and in the first half of the pseudotime bins. We repeated the process of excluding cells 1000 times, resulting in 1000 simulation datasets for each x (Fig. S15f). We applied XCD test to each dataset. The distribution of the p-values for each x is shown in Fig. S15g. The p-vallues became smaller when the group difference becomes larger (i.e. when x increases). Fig. S15h shows the percentage of p-vallues that were smaller than the α=0.05 significance cutoff. When there is no group difference ($x = 0$), less than 5% p-vallues from XCD tests were below 0.05, indicating that XCD test correctly controlled the

Type I error rate. With increasing group difference (i.e., increasing x), the percentage of p-values that were below 0.05 also increased, indicating an increasing power of the XCD test (Fig. S15h).

4 Sample integration and batch correction

The difference between any two samples can be due to true biological differences or unwanted technical differences (e.g. systematic batch effects or random measurement noises). The goal of differential expression analysis is to identify true biological differences. In order to achieve this, there are two main steps in the multiple-sample scRNA-seq differential gene expression analysis.

STEP 1 (sample integration): In this step, one integrates cells from different samples together so that cells of the same type are aligned across samples. This is to make sure that when we compare different samples, we are comparing oranges with oranges (e.g. T cells from patient 1 vs. T cells from patient 2) and apples with apples (e.g. B cells vs. B cells), rather than comparing oranges with apples (e.g. T cells from patient 1 vs. B cells from patient 2). This step is accomplished using existing approaches such as Seurat(CCA) and Harmony. These approaches aim to retain biological differences across cell types, but they remove both biological differences across samples and systematic technical differences such as batch effects. Even though some people call these methods "batch correction" methods, they are actually methods that remove both batch effects and true biological differences across samples to facilitate sample integration. For this reason, we prefer to call them "sample integration" methods rather than "batch correction" methods. These methods may report corrected gene expression values after sample integration (i.e. after removing sample-level differences), but these corrected expression values cannot be directly used to study differential expression across samples (e.g. healthy vs. COVID difference) because all true biological differences between samples are removed together with batch effects.

STEP 2 (differential expression analysis with batch effect correction): After matching cells of the same type across samples, STEP 2 then analyses differential expression using the matched cells. Since one cannot use the corrected expression values from STEP 1 for the reasons explained above, one has to use the original uncorrected gene expression values to perform this analysis. This is also what the authors of Seurat recommended (see the "Identify conserved cell type markers" section: `https://satijalab.org/seurat/articles/integration_introduction.html`, first code chunk: "For performing differential expression after integration, we switch back to the original data"). This is not saying that STEP 1 is not useful. That step is indeed crucial since after STEP 1 we now know which cells are of the same type that we can meaningfully compare across samples. Now for a given type of cell (e.g. T cell), differences in the original gene expression values between different samples contain both true biological difference and technical difference, and one has to separate the true biological difference from the technical difference such as batch effect. For that, one needs to do batch correction. Note that here "batch correction" refers to a procedure that removes batch effects while retaining the true biological differences across samples, which is different from the "sample integration" step in single-cell analysis where both differences are removed.

Lamian supports batch correction (STEP 2) in the differential expression analysis via its regression framework. In its main function `lamian.test()`, users can specify multiple covariates for the samples in the design matrix. This allows one to remove and account for confounding effects across samples such as batch effect, sample's baseline characteristics (e.g. age), genetic background, etc. In the design matrix, each row represents a sample, each column represents a sample covariate, and the matrix entries are values of the sample covariates (except for the first column where all values are equal to 1 to represent the intercept). Users can pass the matrix to the argument `design`, and specify the covariate they are interested in running differential tests in the argument `test.for` as well as the covariate(s) they would like to adjust for as confounding factors in the argument `adjust.for`.

5 Comparisons of Lamian results after Seurat, Harmony and scVI sample integration

Lamian takes integrated scRNA-seq data as input. We assume that the sample integration is done by users. In the main article, `Seurat` was used as the default integration method. We also compared `Seurat` with two other popular sample integration methods `Harmony` and `scVI` using the HCA-BM data. When applying Lamian to `Seurat`-integrated data, pseudotime was inferred using cells' embedding in the principal component space. For `Harmony` and `scVI`, Lamian inferred pseudotime using the top latent variables produced by `Harmony` and `scVI`, respectively. The number of latent variables was selected using the same elbow's method as that in our Lamian analysis based on `Seurat`. Using `Seurat`, `Harmony` and `scVI` all resulted in a tree structure corresponding to the three major hematopoietic differentiation lineages (Figs. S19a,b, S20a,b) and similar branch cell proportion (Figs. S19c, S20c). The genes' pseudotemporal expression patterns fitted by `Seurat+Lamian` showed high Pearson correlation with the fitted expression patterns in the corresponding lineage fitted by `Harmony+Lamian` or `scVI+Lamian` (Figs. S19d,e, S20d,e), indicating that different methods yielded similar pseudotemporal gene expression patterns. Here we calculated the Pearson correlation coefficients to assess the consistency of the Lamian group-level fitted patterns based on `Seurat` and those based on `Harmony` or `scVI` and plotted the distribution of the correlation coefficients

(Fig. S19d, S20d). For XDE analysis, since there were two sample groups (male and female), we also assessed the correlation within each sample group separately (Fig. S19e, S20e). Most correlation coefficients were above 0.5, indicating that similar results were obtained by `Harmony` and `scVI` compared to `Seurat`.

We also checked the overlap between the differential genes identified by `Seurat+Lamian` (FDR<0.05) and the top differential genes identified by `Harmony+Lamian` or `scVI+Lamian`. To compare the TDE genes identified based on `Seurat` and those based on `Harmony` (or `scVI`) outputs, we first ordered genes in the `Harmony` (or `scVI`) gene lists according to decreasing significance, and then calculated the proportion of `Seurat`-reported TDE genes (FDR<0.05) that can be found in the top $100 \times n$ `Harmony` (or `scVI`) genes ($n = 1, 2 \ldots$) (Figs. S19f, S20f: "test results"). As a negative control (i.e. random expectation), the gene lists based on `Harmony` and `scVI` were also permuted to re-calculate the overlap to serve as the baseline (Figs. S19f, S20f: "permuted"). The TDE genes identified by `Harmony+Lamian` or `scVI+Lamian` had a substantial overlap with the TDE genes detected by `Seurat+Lamian` and the overlap was higher than the random expectation. At the 5% FDR cutoff, over 80% of the TDE genes from `Seurat+Lamian` overlapped the TDE genes from `Harmony+Lamian` or `scVI+Lamian` (Figs. S19f, S20f). Similarly, the XDE genes identified by `Seurat+Lamian` and those identified by `Harmony+Lamian` or `scVI+Lamian` also had a substantially higher overlap than random expectation (Figs. S19g, S20g).

6 Analysis of the relationship between cell cycle and cell density

The cell cycle gene lists of S-phase (43 genes) and G2-M-phase (54 genes) were retrieved from Seurat v.3.2.1. The gene list of G1-phase was retrieved from GSEA G1_PHASE geneset (15 genes). For each cell, S-, G2-M- and G1-phase scores were calculated using the average of the normalized log2-transformed gene expression across genes in the corresponding gene lists. In this way, with the cellular pseudotime, the pseudotemporal pattern of the cell cycle scores was obtained. For each sample, Pearson correlation coefficient between cell cycle scores (B-spline fitting to get the same length as the pseudotime window in cell density) and cell density along pseudotime was calculated to assess the association between them. In Fig. S7, the boxplots show the distributions of correlation coefficients for different datasets and tree branches (each data point represents a sample). In all three datasets (HCA-BM, COVID, TB), the cell density had low correlation with all three (S-, G2-M-, and G1-phase) cell cycle scores (the median Pearson correlation coefficients were all around 0), suggesting that the cell density changes along pseudotime in these datasets cannot be explained by cell cycle.

7 Applying other pseudotime methods in `Lamian`

`Lamian` Modules 3 and 4 allow one to use pseudotemporal trajectories generated by other methods to replace `TSCAN` as input for multi-sample differential gene expression and differential cell abundance analysis. Our online user manual (`https://winnie09.github.io/Wenpin_Hou/pages/Lamian.html`) provides a step-by-step tutorial for this using `slingshot` as an example. `Lamian` Modules 1 and 2 which quantify trajectory topology uncertainty and changes currently do not support the other pseudotime methods due to issues such as scalability and other technical complications. For example, `slingshot` is another popular MST-based method similar to `TSCAN` , but it has a more time-consuming principal curve fitting step. It takes `TSCAN` 0.158 seconds to construct the pseudotime (running the main function `TSCANorder()` after model-based clustering) with the HCA-BM data, while `slingshot` needs 857.102 seconds (running the main function `slingshot()` after model-based clustering). To quantify tree uncertainty, we apply the same trajectory construction method repeatedly to bootstrapped datasets. For 100 bootstrap datasets, it will take more than 23 hours for `slingshot` to finish. Increasing the number of bootstraps will further increase the time. Furthermore, each pseudotime method (e.g. `slingshot` , Monocle2, Monocle3, etc.) has its own data structure and trajectory algorithm. Since we are the developer of `TSCAN` , we are able to modify its code to be compatible with `Lamian` . For the other pseudotime methods, directly calling these methods or modifying their code to fit into Lamian is difficult as we do not have complete information on how to access and operate on the relevant data structure (e.g. pseudotemporal trajectory topology) in their code. Implementing them from scratch in `Lamian` is also non-trivial. For this reason, our Modules 1 and 2 currently do not support these methods, but it will be useful to further investigate in the future to find ways to support them in `Lamian` Modules 1 and 2.

8 Options for analyzing branch cell proportion changes

To analyze how branch cell proportion changes with sample covariates in `Lamian` , one can either fit a binomial logistic regression for each branch separately or fit a multinomial logistic regression for all branches jointly.

The advantage of multinomial logistic regression is that it considers the fact that the sum of cell proportions of all branches is equal to one (i.e. different branches are not independent). A disadvantage is that for users who want to study how cell proportion of a specific branch changes with sample covariates, interpretation of its results is less straightforward and sometimes

inconvenient. This can be illustrated using a simple example below. Suppose one compares a treated sample with a control sample, and the samples have three branches. In the control sample, *Sample1*, the cell proportions in three branches are 0.3 (Branch 1), 0.1 (Branch 2) and 0.6 (Branch 3) respectively. Using Branch 3 as the baseline branch, the odds for Branch 1 against Branch 3 (baseline) is $0.3/0.6 = 0.5$. In the treated sample, *Sample2*, the cell proportions in three branches are 0.2 (Branch 1), 0.6 (Branch 2) and 0.2 (Branch 3) respectively. Using Branch 3 as the baseline branch, the odds for Branch 1 against Branch 3 (baseline) is 0.2/0.2 = 1. Comparing the treated sample to the control sample, the odds ratio for Branch 1 against Branch3 (baseline) is $1/0.5 = 2$. In other words, the odds for Branch 1 against baseline Branch 3 is increased in the treated sample (Sample 2) compared to the control sample (Sample 1). However, the actual cell proportion for Branch 1 is decreased from 0.3 in the control sample (Sample 1) to 0.2 in the treated sample (Sample 2). Thus, for users who want to look at the cell proportion changes individually for each branch, looking at the odds ratio (i.e. the regression coefficients) of the multinomial logistic regression does not directly tell one how cell proportion of each branch will change as a sample covariate changes. Although one can convert odds ratios to cell proportions, such a conversion often cannot be processed in real time by human brain when there are many categories or branches, and one will need to use a computer or a calculator for this calculation. This decreases the efficiency in information processing and communication when multiple collaborators sit together to explore data and discuss analysis results in real time. This is analogous to a situation where many people prefer to use log2 over natural logarithm (ln) in microarray or RNA-seq differential gene expression analysis. When one sees a log2 (fold change) = 2, 3, or 4, the human brain can immediately process the information and convert it to the original scale which gives fold change = 4, 8, or 16. But when one sees ln(fold change) = 2, 3 or 4, the human brain cannot quickly convert it to the original scale (i.e. what is the value for fold change = e^2, e^3, or e^4?).

Compared to multinomial logistic regression, the binomial logistic regression is fitted for each branch separately. A disadvantage of this approach is that the dependence of cell proportions from different branches is not considered when branches are modeled separately using different models. However, this approach has the advantage that its results can be conveniently used to study how cell proportion of a specific branch changes with sample covariates. For each branch, the regression models log odds of the underlying true branch cell proportion ($\log(p/(1-p))$ as a function of the sample covariates. A positive regression coefficient means that log odds of cell proportion of the branch increases with the increased value of the corresponding sample covariate. It also means that cell proportion (i.e. p) of the branch increases since log odds is a monotone function of cell proportion. Similarly, a negative regression coefficient means a decrease in cell proportion. Thus, the sign of regression coefficients can be instantaneously translated into the direction of the change (i.e. increase or decrease) of the branch cell proportion.

In summary, binomial logistic regression and multinomial logistic regression each has its own pros and cons. For this reason, we provide both these options in `Lamian` . Users can choose which method to use based on their own needs. For users who want to look at odds ratios in multinomial logistic regression and are experienced at translating odds ratios to cell proportions, they can use multinomial logistic regression. For users interested in how cell proportion of an individual branch changes across samples (i.e. increases or decreases), they can use binomial logistic regression to more conveniently explore the change of each branch in the data.

References

1. Alexa, A. & Rahnenfuhrer, J. topgo: Enrichment analysis for gene ontology. r package version 2.28. 0. *Cranio* (2016).

2. Balaton, B. P., Cotton, A. M. & Brown, C. J. Derivation of consensus inactivation status for x-linked genes from genome-wide studies. *Biol. sex differences* **6**, 1–11 (2015).

3. Byrd, R. H., Lu, P., Nocedal, J. & Zhu, C. A limited memory algorithm for bound constrained optimization. *SIAM J. on scientific computing* **16**, 1190–1208 (1995).

Supplementary Table S1. Comparison of functions provided by Lamian and other pseudotime analysis methods

method	consider variability across multiple samples	XDE test	mean and trend identification in XDE analysis	TDE test	XCD test	TCD test	tree structure uncertainty	pseudotime uncertainty	clustering genes based on temporal pattern	method for pseudotime analysis	version	github
Lamian	yes	yes	yes	yes	yes	yes	yes	yes	yes	yes	0.0.1	https://github.com/Winnie09/Lamian
monocle2	no	no	no	yes	no	no	no	no	yes	yes	2.14.0	http://cole-trapnell-lab.github.io/monocle-release/docs/#clustering-genes-by-pseudotemporal-expression-pattern
monocle3	no	no	no	yes	no	no	no	no	yes	yes	3.0.2.1	https://cole-trapnell-lab.github.io/monocle3/docs/differential/
tradeSeq	no	no	no	yes	no	no	no	no	yes	yes	1.4.0	https://github.com/statOmics/tradeSeq
slingshot (it uses tradeSeq for DE detection)	no	no	no	yes	no	no	no	no	no	yes	1.2.0	https://bioconductor.org/packages/devel/bioc/vignettes/slingshot/inst/doc/vignette.html#identifying-temporally-dynamic-genes
tscan	no	no	no	yes	no	no	no	no	no	yes	1.7.0	https://github.com/zji90/TSCAN
limma	no	no	no	no	no	no	no	no	no	no	3.40.6	http://bioconductor.org/packages/release/bioc/html/limma.html
milo	no	no	no	no	no	yes	no	no	no	no	0.99.8	https://github.com/MarioniLab/miloR
DAseq	no	no	no	no	no	yes	no	no	no	no	1.0.0	https://github.com/KlugerLab/DAseq
pseudotimeDE	no	no	no	yes	no	no	no	yes	no	yes	0.1.0	https://github.com/SONGDONGYUAN1994/PseudotimeDE
condiments	no	yes	no	no	yes	no	no	no	no	yes	v0.99	https://github.com/HectorRDB/condiments
phenopath	no	yes	no	yes	no	no	no	no	no	yes	1.18.0	https://bioconductor.org/packages/release/bioc/html/phenopath.html

Supplementary Table S2. Comparison of computational time and memory usage of different XDE methods.

Computational Time (Hour)

			condiments	Lamian.chisq	Lamian.pm	monocle2TrajTestCorr	phenopath	tradeSeq
	NumberOfSamples	NumberOfCells						
**HCA.Simu**	8	13k	2.961	0.228	2.344	0.1496	14.1616	0.2448
**HCA**	8	13k	4.0775	0.233	2.70877778	0.072333333	14.17666667	0.309333333
**COVID**	161	56k	10.497	1.529	27.12725	0.187	NA	0.578
**TB**	184	337k	NA	8.926	62.605	10.275	NA	NA

Memory (GB)

			condiments	Lamian.chisq	Lamian.pm	monocle2TrajTestCorr	phenopath	tradeSeq
	NumberOfSamples	NumberOfCells						
**HCA.Simu**	8	13k	28.2649872	5.406388	91.9243463	4.8552312	16.2346992	132.3098592
**HCA**	8	13k	28.103318	5.692792	122.82056	7.924004	17.42598667	170.492868
**COVID**	161	56k	77.20384	4.002128	279.96936	28.895608	NA	318.775632
**TB**	184	337k	NA	4.89568	243.04102	43.28332	NA	NA
