# Supplementary material for: A statistical framework for differential pseudotime analysis with multiple single-cell RNA-seq samples
Source: Nat Commun. 2023 Nov 10;14:7286. doi: 10.1038/s41467-023-42841-y (PMC10638410; doi:10.1038/s41467-023-42841-y)
Supplement: Supplementary file 3 — Editorial Assessment Report [file 41467_2023_42841_MOESM3_ESM.pdf]

nature portfolio

GUIDED OPEN ACCESS

nature methods
nature communications
communications biology

Contents of this report

1. **Manuscript details**: overview of your manuscript and the editorial team.
2. **Review synthesis**: summary of the reviewer reports provided by the editors.
3. **Editorial recommendation**: personalised evaluation and recommendation from all 3 journals.
4. **Annotated reviewer comments**: the referee reports with comments from the editors.
5. **Open research evaluation**: advice for adhering to best reproducibility practices.

About the editorial process

Because you selected the **Nature Portfolio Guided Open Access option**, your manuscript was assessed for suitability in three of our titles publishing high-quality work across the spectrum of methods research: *Nature Methods, Nature Communications*, and *Communications Biology*. More information about Guided Open Access can be found here.

Collaborative editorial assessment

Your editorial team discussed the manuscript to determine its suitability for the Nature Portfolio Guided OA pilot. Our assessment of your manuscript takes into account several factors, including whether the work meets the **technical standard** of the Nature Portfolio and whether the findings are of **immediate significance** to the readership of at least one of the participating journals in the Nature Portfolio Guided Open Access methods cluster.

Peer review

Experts were asked to evaluate the following aspects of your manuscript:

1. **Novelty** in comparison to prior publications;
2. **Likely audience** of researchers in terms of broad fields of study and size;
3. **Potential impact** of the study on the immediate or wider research field;
4. **Evidence** for the claims and whether additional experiments or analyses could feasibly strengthen the evidence;
5. **Methodological detail** and whether the manuscript is reproducible as written;
6. Appropriateness of the literature review.

Editorial evaluation of reviews

Your editorial team discussed the potential suitability of your manuscript for each of the participating journals. They then discussed the revisions necessary in order for the work to be published, keeping each journal's specific editorial criteria in mind.

Journals in the Nature portfolio will support authors wishing to transfer their reviews and (where reviewers agree) the reviewers' identities to journals outside of Springer Nature.

If you have any questions about review portability, please contact our editorial office at guidedOA@nature.com.

nature portfolio

GUIDED OPEN ACCESS

nature methods | nature communications | communications biology

Manuscript details

Tracking number	Submission date	Decision date
GUIDEDOA-21-00187	11 July 2021	15 October 2021

**Title**	A statistical framework for differential pseudotime analysis with multiple single-cell RNA-seq samples	**Corresponding author**	Hongkai Ji<br>**Affiliation:** Johns Hopkins Bloomberg School of Public Health
**Preprint information**	There is a preprint of this manuscript posted at bioRxiv	**Peer review type**	Single-blind

Editorial assessment team

**Primary editor**	**Home Journal:** *Nature Methods*, ORCID: 0000-0002-6050-0424<br>**Email:** lin.tang@nature.com
**Editorial team members**	**Doaa Megahed**, *Nature Communications*, ORCID: 0000-0002-3455-2992<br>**George Inglis**, *Communications Biology*, ORCID: 0000-0002-9069-5242
**About your primary editor**	Lin Tang obtained his Ph.D. in Computational Biology at the CAS-MPG Partner Institute for Computational Biology, Chinese Academy of Sciences, studying transcriptome evolution. He was an Associate Editor for *Nature Communications* beginning in November 2016, and joined *Nature Methods* as a Senior Editor in September 2019. He handles genomics and computational methods for the journal.

Editorial assessment and review synthesis

**Editor's summary and assessment**	Hou et al. develop the Lamian computational framework for differential pseudotime analysis using multiple scRNA-seq samples. Lamian evaluates uncertainty of tree branches and differential topology, and performs various trajectory differential tests. The method is demonstrated using simulated and real single-cell datasets.<br><br>The editors felt the topic of differential pseudotime analysis interesting, but, in light of the reviewer comments, think the conceptual advances of the method may not be sufficient to meet the criteria for *Nature Methods* or *Nature Communications*.
**Editorial synthesis of reviews**	While our reviewers find this work of potential interest, a number of key concerns were raised, including novelty and comparison to existing approaches, biological insights and applications, as well as other important conceptual, technical and presentation issues. Altogether, these concerns prohibited further consideration by *Nature Methods* or *Nature Communications*.<br><br>At a minimum, a revision for *Communications Biology* should include further benchmarking to alternative methods, justification of steps in the Lamian analysis, and, if feasible, a case study demonstrating how Lamian can provide new biological insight.

Editorial recommendation

nature methods — **Revision not invited**

The conceptual advance demonstrated is not sufficient for publication in *Nature Methods*.

nature communications — **Revision not invited**

Unfortunately, the reviewers raise several concerns that bring into question the extent of novelty and utility of the method compared to others in this space.

communications biology — **Major Revisions**

In light of the supportive feedback from Reviewers #1 and #3, we would be interested in considering a revised manuscript that includes additional benchmarking, justification of steps in the Lamian analysis, and, if feasible, a case study demonstrating how Lamian can provide new biological insight.

Next Steps

Recommendation Summary:

- **Option 1:** Revise for consideration at *Communications Biology*.
- **Option 2:** Revise for consideration elsewhere.

See the previous page for details. ***Nature Methods*** *and* ***Nature Communications*** *can no longer consider the manuscript due to concerns about the conceptual advance, as well as the generalizability and utility of Lamian in comparison to existing alternative methods.*

Revision

If you would like to follow our recommendation, please upload the revised manuscript, along with your point-by-point response to the reviewers' reports and editorial advice **using the link provided in the decision letter**. Should you need assistance with our manuscript tracking system, please contact Adam Lipkin, our Nature Portfolio Guided OA support specialist, at guidedOA@nature.com.

Revision checklist

1. Cover letter, stating to which journal you are submitting
2. Revised manuscript
3. Point-by-point response to reviews
4. Updated **Reporting Summary** and **Editorial Policy Checklist**
5. Supplementary materials (if applicable)

Submission elsewhere

Within the Nature Portfolio

Springer Nature provides authors with the ability to transfer a manuscript within the Nature Portfolio, without the author having to upload the manuscript data again. To use this service, please **request a transfer link** from guidedOA@nature.com.

Note that any decision to opt in to *In Review* at the original journal is not sent to the receiving journal on transfer. You can opt in to *In Review* at receiving journals that support this service by choosing to modify your manuscript on transfer.

To a journal outside of Nature Portfolio

We can share the reviews with another journal outside of the Nature Portfolio if requested. You will need to request that the receiving journal office contacts us at guidedOA@nature.com. We have included editorial guidance below in the reviewer reports and open research evaluation to aid in revising the manuscript for publication elsewhere.

Annotated reviewer reports

The editors have included some additional comments on specific points raised by the reviewers below, to clarify requirements for publication in the recommended journal(s). However, please note that all points should be addressed in a revision, even if an editor has not specifically commented on them.

**Reviewer #1**	
**Reviewer #1**	This reviewer has not chosen to waive anonymity. The reviewer's identity can only be shared with representatives of an established journal editorial office.
**Reviewer #1 expertise**	This reviewer has expertise in single-cell analyses and computational biology
**Editor's comments about this review**	This reviewer has raised important technical concerns on different aspects of the method, analysis, and comparison with other methods.

**Reviewer #1 comments**	
**Overview**	In this paper, the authors propose a statistical framework, Lamian, for multi-sample pseudotime differential analysis. Lamian includes the following major modules:<br>1. Quantifying the uncertainty of the tree branches by bootstrap;<br>2. Differential tree topology between two conditions by comparing branch proportions (defined by numbers of cells assigned to branches);<br>3. A gene's differential expression (DE) analysis along a pseudotemporal trajectory / a branch, including TDE (if a gene has expression changes along the trajectory) or XDE (if a gene has the same expression dynamics between two conditions);<br>4. Differential cell density (CD) analysis along a pseudotemporal trajectory / a branch (TCD and XCD).<br>The authors applied Lamian to two real scRNA-seq datasets to demonstrate its effectiveness in applications.<br>Overall, I think this article is well-written and the Lamian framework is easy to understand. One key novelty is that the authors used the mixed-effect like models to address multi-sample variation. However, I have the following questions and suggestions about the statistical methods used in Lamian.

Specific comments		
**#**	**Reviewer comment**	**Editorial comment**
**1**	The Lamian relies on TSCAN for pseudotime inference. This leads to two questions. First, can Lamian be used as a downstream pipeline for other popular pseudotime inference methods, e.g., Slingshot and Monocle3? Second, for the comparison with two existing DE methods (limma and tradeSeq), do all DE methods use the same input pseudotime? If not, is it possible that the quality of pseudotime inference affects the DE performance so that the comparison is not completely fair?	**It is important to clarify these features for further consideration at *Communications Biology*.**
**2**	A drawback of Lamian is that it does not account for the uncertainty in pseudotime inference because it only applies the bootstrap to the pseudotime values of all cells. In other words, the bootstrap only captures the randomness in sampling cells, not the randomness in the inference pseudotime values of all cells.	**This and other limitations of the method should be clearly discussed in the revision.**
**3**	Because of **Point #2**, the permutations used in the likelihood-ratio tests (LRTs) for XDE and TDE are not necessary because the null distribution only captures the randomness in sampling cells. Hence, the authors should be able to use the theoretical, asymptotic null distribution of the LRT statistic and greatly reduce the computational time required by permutations.	
**4**	A computational issue related to **Point #2** is that the authors used the kernel density estimate (KDE) of the permuted LRT statistic values as the null distribution. However, KDE relies on a pre-specified kernel bandwidth, which is automatically chosen by the R function for every gene but not necessarily appropriate for some genes. This computational instability should be avoided by replacing the permutation approach by the theoretical null distribution as I suggested in **Point #2**.	
**5**	Module 2 uses the two-sample t test to compare branch proportions between two conditions. The t test is inappropriate for two reasons: (1) branch proportions are not normally distributed; (2) proportions of different	

	branches are not i.i.d.	
6	A limitation of Module 2 is: what if two conditions have different topologies, e.g., the two conditions have difference single branches. In this case, how can Lamian tell that the two single branches are not the same?	
7	**Figure 4h** suggests that the simulation for power comparison and FDR control was not realistic. The ROC curve is too perfect. I suggest that the authors add a more realistic simulation study for benchmarking Lamian against limma and tradeSeq.	**This point would be necessary for further consideration at *Communications Biology*.**
8	In Methods, why do equations (2) and (4) share the same variance \sigma_s^2? Equation (4) does not seem to need \sigma_s^2.	
9	**Table S1** listed several existing methods, but these methods were not cited in the main text. The authors should mention these methods in the main text and add their citations.	**For the sake of reproducibility, please cite these Methods and list any relevant version information.**
10	A right parenthesis in line 27 on page 7 should be removed.	
11	**Regarding impact:** *Nature Communications*	**While we appreciate the reviewer's input, we must emphasize that decisions regarding publication are solely made by the editors.**
12	**Regarding strength of claims:** Please see my comments under the overall significance.	
13	**Regarding reproducibility:** I didn't run the code, but the writing was overall clear.	

Reviewer #2	
Reviewer #2	This reviewer has not chosen to waive anonymity. The reviewer's identity can only be shared with representatives of an established journal editorial office.
Reviewer #2 expertise	This reviewer has expertise in single-cell analyses and computational biology.
Editor's comments about this review	This reviewer has raised concerns on novelty, biological insights and applications, as well as other important conceptual, technical and presentation issues. This reviewer highlights the need to better distinguish Lamian from alternative methods both in the text and through additional benchmarking.
**Reviewer #2 comments**	
Overview	In this work, Wenpin Hou, et. al. reported a statistical framework, Lamian, that builds on top of other approaches to perform comprehensive differential pseudotime analyses of scRNA-seq datasets with consideration of multiple samples. In particular, Lamian deal with multi-sample datasets and is able to identify changes in differential genes along pseudotemporal trajectory that are related to various sample covariates, as well as to detect the associated changes in gene expression, cell density, and topology.

Specific comments

#	Reviewer comment	Editorial comment
**Major Comments:**		
1	**Regarding impact:** *Scientific Reports*; I don't think this will influence thinking in the field.	**As before, all decisions regarding publication are made by editors.**
2	The authors argue that comparing pseudotemporal dynamics across multiple samples is lacking and claim lamina is one of the first model to do so. This claim, however, is rather weak and should be tuned down or backed up much better. The author should do a more inclusive review or summary of existing tools for multi-sample pseudotime analysis to support this claim (for example, providing a supplementary table to summarize existing pseudotime analyses tools and how lamina fills	**Further benchmarking and reporting of Lamian's advantages over existing methods would be necessary for consideration at *Communications Biology*.**

	this gap).<br><br>For example, Kieran R Campbell (https://www.nature.com/articles/s41467-018-04696-6) has published papers on differential pseudotime analyses with covariates. In addition, the trajectory-conditioned test from Monocle 2 (Fig 2 of https://www.ncbi.nlm.nih.gov/pmc/articles/PMC5764547/) performs differential analyses to identify genes that change under different genetic backgrounds (samples) along pseudotime trajectory.<br><br>Fundamentally, inclusion of the sample covariate when performing a generalized linear regression is a rather conventional practice in biostatistics and thus the novelty of this approach is limited.	
**3**	Furthermore, different samples may lead to strong batch effects that are not biologically relevant. Thus, it is important to discern the real biological difference and the technical differences between different samples. The author relies on existing approaches to remove batches before downstream differential analyses but it is worth discussing this issue. Another possibility is to avoid the batch effect issue by focusing on analyzing datasets that are generated from the same batch but still involve different samples (for example, including drug treatment, genetic perturbation, etc.).	**Discussion of this point would be necessary for further consideration at *Communications Biology*.**
**Minor Comments:**		
**4**	**Page 1, Line 20:** the author mentioned spatial dynamics, however the entire paper doesn't really touch analyses of this type of data and I would suggest removing this to avoid spatial transcriptomics bandwagon.	
**5**	**Page 2:** The first step of Lamian uses TSCAN and bootstrapping for pseudotime and batch certainty detection. There are obviously many superb alternatives, including principal graph, diffusion pseudotime, etc., than TSCAN for pseudotime analysis and it is important to explain why this choice was used (even if this is something that is related to your lab's previous research). In addition, Ruslan Soldatov, et.al previously reported using bootstrapping of the developmental tree construction with simplePPT to estimate the certainty of the tree	

	structure. It may be interesting to compare your approach with this method (https://www.science.org/doi/10.1126/science.aas9536?url_ver=Z39.88-2003&rfr_id=ori:rid:crossref.org&rfr_dat=cr_pub_0pubmed)	
6	**Page 3:** Some information about the schematic is not very clear. For example, what is the meaning of 0.3 (0.2) for example, in the section related to cell proportion in a branch of the panel b (mean and s.d.?) Similarly, simple explanations of the meaning of the equations in panel C needs to be spelled out in the schematic.	
7	**Page 4:** Overall, the author needs to justify why the HCA-BM dataset was used for the analyses and why comparing the male and female is meaningful and interesting at all. For example, the analysis in 2.2.2 seems quite strange because you won't ever expect the hematopoiesis to differ across men and women.	
8	In **2.2.3,** the author shows that decreasing the number of cells decreased the detection rate for myeloid branch.<br><br>It will be interesting to check whether this low detection rate is biologically relevant or whether it is purely driven by uneven capture of cell number for the myeloid lineage.	
9	**Page 5:** Following gene short name convention, the author may need to go through the paper carefully to italicize all gene names (for example CD14 to CD14, etc.). Importantly, the example genes shown here are less informative as most of them are CD markers which are reported previously based on FACS data (protein level). It will be more relevant to showcase known transcription factors or other markers which have a known high RNA expression level for each particular lineage.	
10	Lastly, some of the enrichment analysis doesn't make the perfect sense, for example, why "as HSCs differentiate to the erythroid lineage, the TDE genes with initially 4 high expression but low expression at the end are enriched in CD8-positive, alpha-beta T cell activation"? What we expect is that those genes should be enriched in HSC stemness maintenance related pathways. Similarly the	

	"platelet degranulation" doesn't make a lot of sense.	
11	**Page 7:** The number of the XDE genes detected are very few and mostly related to the X/Y-chromsome, can you find any meaningful gene that are related to hematopoesis while also have sex-difference. Basically I wonder whether what you identified are simply genes that differ between male and female but have nothing to do with hematopoiesis. This boils down again to whether it is meaningful to even perform the differential analyses between different sex for the hematopoesis.	
12	The author didn't obtain any biological insights with the cell density analyses. Can the author find a dataset to demonstrate the relevance of this approach?	**While we would strongly encourage you to analyze datasets for new biological insight, this point would not be required for *Communications Biology*.**
13	**Page 9:** The authors try to provide some biological insights in the final section on SARS-Covid2 infection but their conclusions are too tentative which provide little advance the Covid-19 biology and it is not clear whether their work on the subject is to drive the Covid bandwagon to their method.	
14	**Regarding reproducibility:** Great, as it was provided as an associated package.	

Reviewer #3	
**Reviewer #3**	This reviewer has not chosen to waive anonymity. The reviewer's identity can only be shared with representatives of an established journal editorial office.
**Reviewer #3 expertise**	This reviewer has expertise in single-cell analyses and computational biology.
**Editor's comments about this review**	This reviewer has raised important technical concerns on different aspects of the method and analysis.

Reviewer #3 comments	
**Overview**	Hou et al presented a comprehensive statistical framework Lamian for pseudo-time analysis of single cell RNA-seq data from multiple samples with various conditions. The main functions of Lamian include preprocessing, tree structure inference, evaluating topology difference, identifying differential gene along psuedotime or between different conditions. They used both simulations and real scRNAseq data, including COVID-19 patients with different disease severity levels, to demonstrate the advantages of Lamian over competing software such as tradeSeq. The paper is well written and the statistical model is rigorous. It adds novel tools to the crowded trajectory analysis field, which mainly focuses on single sample analysis. I have the following comments to strengthen the paper.

Specific comments

#	Reviewer comment	Editorial comment
**Major Comments:**		
**1**	The authors described the input for Lamian includes a low-dimensional space representation of scRNA-seq data from multiple samples after batch effect correction (**page 2, line 18-20**), and listed candidate methods such as Seurat and Harmony. However, in the paper the authors conducted all analyses using Seurat approach (**Section 4.2, page14, line 20**), but didn't try to illustrate Lamian's performance with input from alternative methods, for example Harmony. It is important to provide some practical	**This point would be necessary for further consideration at *Communications Biology*.**

	guidance to users about which batch effect methods to use under different conditions (e.g., different tissue types), and how the result differs. Another popular batch effect method that should fit Lamian is scVI (https://www.nature.com/articles/s41592-018-0229-2). I suggest the authors try both Harmony and scVI in addition to Seurat for this data processing step to see if the result differ much, and discuss more about it.	
2	For HCA-BM data, the authors mentioned they identified 6 cell clusters after applying TSCAN to the harmonized bone marrow data. However, as a complex tissue type, people can usually identify 10-20 or even more cell subpopulations. Is it an over-simplification of the problem by setting the number of cell clusters as 6 here? In addition, can the authors further instruct if Lamian can accommodate a large number of cell clusters, which form a tree with a large number of branches?	**It would be particularly useful to comment on the scalability of Lamian.**
3	As for computational burden, the authors mentioned Lamian is computationally tractable. However, with the example of HCA bone marrow dataset (32,819 cells and 8 samples), it requires 4.1 hours with 25 CPUs and 163 GB RAM (page 11, line 19-21), which doesn't seem much friendly to users, and cannot be handled with a laptop. Since Lamian is designed for multi-batch data, which could scale up to 100K or 1M cells coming from a larger number of samples, will Lamian still work? Some discussion on computational complexity is needed.	
4	This paper lacks a comparison with a recent competing method called condiments (https://www.biorxiv.org/content/10.1101/2021.03.09.433671v1).	**Benchmarking to condiments would be necessary for further consideration at *Communications Biology*.**
5	The authors showed "decreasing the number of cells decreased the detection rate". This might be a limitation: as different datasets may vary in cell numbers, how can we believe the detection rate would be high enough to be different from random testing, especially when the dataset has limited number of cells?	
6	In the validation of XDE test (P7), the author mentioned the identified XDE genes are enriched in sex	

	chromosomes, as the argument to show their method can accurately captured the DEGs between male and female. Did the author check whether the identified XDE genes on ChrX are the known genes escaping X-inactivation? If yes, this can further support the authors' results.	
**7**	XCD test is not very attractive in my opinion, just as the author mentioned "the change can be due to technical sampling bias or real biology". In addition, the changes in cell cycle status or proliferation rate may be a major reason leading to the changes in cell density along pseudotime. Can the authors check whether there are any relationships between their detected changes in cell density and the distribution of cell cycle status along pseudotime?	
**Minor Comments:**		
**8**	In **Section 2.1.3**, the authors mentioned unsupervised k-means clustering is applied to DE genes (page 4, line 1). Does the result differ much if other clustering methods are used, such as Louvain or GMM?	
**9**	In **Section 4.1** Data (**page 14, lines 5-9**), the authors stated the raw HCA-BM data consist of 290,861 cells, but after screening, the final data for analysis consist of only 32,819 cells, which means nearly 90% of cells are screened out. The authors should justify why such a strict screening is necessary and provide descriptive plots to illustrate the choice of 5,000 reads, 1,000 expressed genes and 10% mitochondrial gene expression are reasonable thresholds.	
**10**	It is unclear what Lamian stands for as an abbreviation. Some explanation is needed. Both Lamian (majority) and Lamian were used in the text.	
**11**	**Regarding impact:** *Nature Methods* is appropriate for the manuscript given the novelty of the statistical approach, the broad application of the paper, and convincing biological examples.	**As before, all decisions regarding publication are solely made by the editors.**
**12**	**Regarding reproducibility:** All data are publicly available. Code has been deposited to GitHub.	

Open research evaluation

Data availability

Please add a Data Availability statement. Please ensure that your Data Availability statement includes accession details for deposited data, mentions where Source data can be found, and states that all other data are available from the corresponding author (or other sources, as applicable) on reasonable request. More information about our data availability policy can be found here: https://www.nature.com/nature-portfolio/editorial-policies/reporting-standards#availability-of-data

See here for more information about formatting your Data Availability Statement: http://www.springernature.com/gp/authors/research-data-policy/data-availability-statements/12330880

This journal strongly supports public availability of data and custom code associated with the paper in a persistent repository where they can be freely and enduringly accessed or as a supplementary data file when no appropriate repository is available. If data and code can only be shared on request, please explain why in your Data Availability Statement, and also in the correspondence with your editor.

For more information, please refer to https://www.nature.com/nature-research/editorial-policies/reporting-standards#availability-of-data

Please ensure that datasets deposited in public repositories are now publicly accessible, and that accession codes or DOI are provided in the "Data Availability" section. As long as these datasets are not public, we cannot proceed with the acceptance of your paper. For data that have been obtained from publicly available sources, please provide a URL and the specific data product name in the data availability statement. Data with a DOI should be further cited in the methods reference section.

Data citation

Please cite (within the main reference list) any datasets stored in external repositories that are mentioned within their manuscript. For previously published datasets, we ask that you cite both the related research article(s) and the datasets themselves. For more information on how to cite datasets in submitted manuscripts, please see our data availability statements and data citations policy: https://www.nature.com/documents/nr-data-availability-statements-data-citations.pdf

Citing and referencing data in publications supports reproducible research, by increasing the transparency and provenance tracking of data generated or analysed during research. Citing data formally in reference lists also helps facilitate the tracking of data reuse and may help assign credit for individuals' contributions to research. A number of Springer Nature imprints are signatories of the Joint Declaration on Data Citation Principles, which stress the importance of data resources in scientific communication.

Code availability and citation

Please include a statement under the heading "Code Availability", indicating whether and how the custom code/software reported in your study can be accessed, including any restrictions to access. This section should also include information on the versions of any software used, if relevant, and any specific variables or parameters used to generate, test, or process the current dataset. Code availability statements should be provided as a separate section after the Data Availability section.

Upon publication, Nature Portfolio journals consider it best practice to release custom computer code in a way that allows readers to repeat the published results. Code should be deposited in a DOI-minting repository such as Zenodo, Gigantum or Code Ocean and cited in the reference list following the guidelines described in our policy pages (see link below). Authors are encouraged to manage subsequent code versions and to use a license approved by the open source initiative.

Full details about how the code can be accessed and any restrictions must be described in the Code Availability statement.

See here for more information about our code availability policies: https://www.nature.com/nature-portfolio/editorial-policies/reporting-standards#availability-of-computer-code

We also provide a Code and Software submission checklist that you may find useful: https://www.nature.com/documents/nr-software-policy.pdf

Please note: because of advanced features used in this form, you must use Adobe Reader to open the documents and fill it out.

Ethics

Please reiterate any relevant ethics information or clarify informed consent from the source datasets used in this study.

Reproducibility

Please state in the legends how many times each experiment was repeated independently with similar results. This is needed for all experiments, but is particularly important wherever results from representative experiments (such as micrographs) are shown. If space in the legends is limiting, this information can be included in a section titled “Statistics and Reproducibility” in the methods section.

Statistics

Wherever statistics have been derived (e.g. error bars, box plots, statistical significance) the legend needs to provide and define the n number (i.e. the sample size used to derive statistics) as a precise value (not a range), using the wording "n=X biologically independent samples/animals/cells/independent experiments/n= X cells examined over Y independent experiments" etc. as applicable.

Legends requiring revision: Figure 2d.

Please note that statistics such as error bars significance and p values cannot be derived from n<3 and must be removed in all such cases.

We strongly discourage deriving statistics from technical replicates, unless there is a clear scientific justification for why providing this information is important. Conflating technical and biological variability, e.g., by pooling technically replicates samples across independent experiments is strongly discouraged. (For examples of expected description of statistics in figure legends, please see the following https://www.nature.com/articles/s41467-019-11636-5 or https://www.nature.com/articles/s41467-019-11510-4).

All error bars need to be defined in the legends (e.g. SD, SEM) together with a measure of centre (e.g. mean, median). For example, the legends should state something along the lines of "Data are presented as mean values +/- SEM" as appropriate.

All box plots need to be defined in the legends in terms of minima, maxima, centre, bounds of box and whiskers and percentile.

The figure legends must indicate the statistical test used. Where appropriate, please indicate in the figure legends whether the statistical tests were one-sided or two-sided and whether adjustments were made for multiple comparisons.

For null hypothesis testing, please indicate the test statistic (e.g. F, t, r) with confidence intervals, effect sizes, degrees of freedom and P values noted.

Please provide the test results (e.g. P values) as exact values whenever possible and with confidence intervals noted.

Legends requiring revision:

1. Please indicate the statistical test used for data analysis and where appropriate, please specify whether it was one-sided or two-sided and whether adjustments were made for multiple comparisons, in the legends of Figure 5d, Supplementary Figures 3a-d, 4c, 4e, 6c-d, 6g-h.
2. Please note that the exact p value should be provided, when possible, in the legends of Figure 5d, Supplementary Figures 3a-d, 4c, 4e

Data presentation

Please ensure that data presented in a plot, chart or other visual representation format shows data distribution clearly (e.g. dot plots, box-and-whisker plots). When using bar charts, please overlay the corresponding data points (as dot plots) whenever possible and always for n ≤ 10. (Please see the following editorial for the rationale behind this request and an example https://www.nature.com/articles/s41551-017-0079).

Please note that data presentation has to be revised to comply with our policy in Figure 2d.

Other notes

We have included as an attachment to the decision letter a version of your Reporting Summary with a few notes. This is mainly for your information, but we hope it is helpful when preparing your revised manuscript. If you decide to resubmit the manuscript for further consideration, please be sure to include an updated Reporting Summary.
