# Supplementary material for: A statistical framework for differential pseudotime analysis with multiple single-cell RNA-seq samples
Source: Nat Commun. 2023 Nov 10;14:7286. doi: 10.1038/s41467-023-42841-y (PMC10638410; doi:10.1038/s41467-023-42841-y)
Supplement: Supplementary file 4 — Inventory of Supporting Information [file 41467_2023_42841_MOESM4_ESM.docx]

**Inventory of Supplementary Information**

Supplementary Information contains the following:

**Supplementary Figures** --- Supplementary Figures S1-S21.

**Supplementary Notes** --- Supplementary Notes of methods.

**Supplementary Table S1** -- Comparison of functions provided by Lamian and other pseudotime analysis methods.

**Supplementary Table S2** -- Comparison of computational time and memory usage of different XDE methods.
